# Flow-induced glycocalyx formation and cell alignment of HUVECs compared to iPSC-derived ECs for tissue engineering applications

**DOI:** 10.3389/fcell.2022.953062

**Published:** 2022-09-05

**Authors:** Marcus Lindner, Anna Laporte, Laura Elomaa, Cornelia Lee-Thedieck, Ruth Olmer, Marie Weinhart

**Affiliations:** ^1^ Institute of Chemistry and Biochemistry, Freie Universität Berlin, Berlin, Germany; ^2^ Institute of Physical Chemistry and Electrochemistry, Leibniz Universität Hannover, Hannover, Germany; ^3^ Institute of Cell Biology and Biophysics, Leibniz Universität Hannover, Hannover, Germany; ^4^ Leibniz Research Laboratories for Biotechnology and Artificial Organs (LEBAO), Department of Cardiothoracic, Transplantation and Vascular Surgery, Hannover Medical School, Biomedical Research in Endstage and Obstructive Lung Disease (BREATH), Member of the German Center for Lung Research (DZL), Hannover, Germany; ^5^ REBIRTH–Research Center for Translational Regenerative Medicine, Hannover Medical School, Hannover, Germany

**Keywords:** glycocalyx model, homogeneous shear stress, laminar flow, mechanotransduction, cellular directionality, peripheral blood mononuclear cell adhesion assay, thermoresponsive coating, cell sheet engineering

## Abstract

The relevance of cellular *in vitro* models highly depends on their ability to mimic the physiological environment of the respective tissue or cell niche. Static culture conditions are often unsuitable, especially for endothelial models, since they completely neglect the physiological surface shear stress and corresponding reactions of endothelial cells (ECs) such as alignment in the direction of flow. Furthermore, formation and maturation of the glycocalyx, the essential polysaccharide layer covering all endothelial surfaces and regulating diverse processes, is highly dependent on applied fluid flow. This fragile but utterly important macromolecular layer is hard to analyze, its importance is often underestimated and accordingly neglected in many endothelial models. Therefore, we exposed human umbilical vein ECs (HUVECs) and human induced pluripotent stem cell-derived ECs (iPSC-ECs) as two relevant EC models in a side-by-side comparison to static and physiological dynamic (6.6 dyn cm^−2^) culture conditions. Both cell types demonstrated an elongation and alignment along the flow direction, some distinct changes in glycocalyx composition on the surface regarding the main glycosaminoglycan components heparan sulfate, chondroitin sulfate or hyaluronic acid as well as an increased and thereby improved glycocalyx thickness and functionality when cultured under homogeneous fluid flow. Thus, we were able to demonstrate the maturity of the employed iPSC-EC model regarding its ability to sense fluid flow along with the general importance of physiological shear stress for glycocalyx formation. Additionally, we investigated EC monolayer integrity with and without application of surface shear stress, revealing a comparable existence of tight junctions for all conditions and a reorganization of the cytoskeleton upon dynamic culture leading to an increased formation of focal adhesions. We then fabricated cell sheets of EC monolayers after static and dynamic culture *via* non-enzymatic detachment using thermoresponsive polymer coatings as culture substrates. In a first proof-of-concept we were able to transfer an aligned iPSC-EC sheet to a 3D-printed scaffold thereby making a step in the direction of vascular modelling. We envision these results to be a valuable contribution to improvements of *in vitro* endothelial models and vascular engineering in the future.

## 1 Introduction

Mimicking the physiological cellular niche by translation and adaption of chemical and physical cues has become a valuable tool in *in vitro* primary and stem cell culture as well as tissue engineering (Metallo et al., 2008; Barthes et al., 2014; Chatterjee et al., 2021). In the engineering and biofabrication of blood vessels, functional endothelial cells (ECs) are of utmost importance as they form the inner lining of all blood vessel walls (Devillard and Marquette, 2021). In addition to tight junctions, the luminally expressed, dense macromolecular layer consisting of glycoproteins, proteoglycans (PGs), glycosaminoglycans (GAGs) as well as soluble plasma factors, known as the glycocalyx, contributes significantly to the endothelial barrier function. This net negatively charged polysaccharide-rich layer is linked to the EC surface on the one hand *via* glycoproteins such as selectins, integrins, and immunoglobulins and on the other hand by some types of PGs which are anchored in the EC membrane. The main structural feature of membrane-bound as well as secreted PGs is the presentation of GAG side-chains along the PG core protein. Within the glycocalyx the most abundant PG-bound GAG is heparan sulfate (HS) with 50%–90% frequency, followed by chondroitin sulfate/dermatan sulfate (CS/DS) and the non-PG-bound GAG hyaluronic acid (HA) (Oohira et al., 1983; Ihrcke et al., 1993; Reitsma et al., 2007; Cosgun et al., 2020). Glycocalyx thickness varies between species as well as different types of blood vessels and is strongly dependent on the applied fluid-flow induced surface shear stress, ranging from ∼0.5 µm in capillaries up to 4–5 µm in carotid arteries (Gouverneur et al., 2006a; Gouverneur et al., 2006b; Reitsma et al., 2007; Tarbell et al., 2014). From a functional point of view, the glycocalyx plays a major role in regulating the vascular permeability, controlling the endothelial interactions with blood cells as well as signaling. Additionally, it acts as a protective barrier for the endothelium against blood flow-induced shear stress, while simultaneously sensing mechanical forces at the cell surface communicating it into the interior (Reitsma et al., 2007; Weinbaum et al., 2007; Curry and Adamson, 2012; Fels and Kusche-Vihrog, 2020). Important mechanotransducers on the cell surface are sialic acids as end groups of glycoprotein polysaccharide side chains (Psefteli et al., 2021), HS (Florian et al., 2003; Ebong et al., 2014) and HA (Mochizuki et al., 2003). As a result, ECs elongate and align along the flow direction *in vivo* as well as *in vitro* (Thoumine et al., 1995; Steward et al., 2015).

The importance of the glycocalyx for endothelial function has long been underestimated and quantitative studies are challenging due to its highly dynamic and fragile nature (Schött et al., 2016; Möckl, 2020). Especially *in vitro* cultivation conditions can have a major impact on its status, with conventional static cell culture leading to unphysiological glycocalyx thicknesses and compositions (Potter and Damiano, 2008; Chappell et al., 2009).

A commonly used cellular model for *in vitro* studies of the endothelial barrier are human umbilical vein endothelial cells (HUVECs). As robust and accessible primary cells they hold several advantages over immortalized cell lines such as contact inhibition upon reaching confluency and especially physiological characteristics of the human vascular endothelium including *in vivo*-like responses to a variety of stimuli. However, the donor-to-donor variability as well as a strongly limited culture time urge the need for alternatives (Jaffe et al., 1973; Cao et al., 2017; Medina-Leyte et al., 2020). Particularly ECs derived from human induced pluripotent stem cells (iPSC-ECs) are a promising candidate to fill this gap. Several successful protocols for differentiation have been established so far (Orlova et al., 2014a; Patsch et al., 2015; Olmer et al., 2018), generating iPSC-ECs featuring various properties of a vascular endothelium, for example, regarding marker expression, phenotype or response to mechanical or chemical stimuli, representing a potential cell source for biologization of blood contacting surfaces (Pflaum et al., 2021). Furthermore, use of iPSC-ECs yields the potential to generate patient-specific or disease-related vascular lineages and of unlimited cell supply originating from the same donor (Jang et al., 2019; Kennedy et al., 2021). However, the evaluation of iPSC-ECs’ eligibility as an alternative to primary cells in models of the vascular endothelium appears incomplete. Studies suggest that iPSC-derived cells could be less mature than primary cells, exemplary in being shear-naïve (Sivarapatna et al., 2015; Tiemeier et al., 2019; Kennedy et al., 2021). Moreover, detailed analyses on the glycocalyx condition in iPSC-ECs are needed.

As side-by-side comparisons between HUVECs and iPSC-ECs under different culture conditions are still rare, in the present study we cultured these EC types under static as well as dynamic conditions with particular focus on the glycocalyx. Under dynamic conditions a homogenous defined shear stress (6.6 dyn cm^−2^) was applied to the cell surface, thereby mimicking physiological fluid flow in veins and capillaries (Ballermann et al., 1998; Paszkowiak and Dardik, 2003). The effects on cellular elongation and alignment as well as glycocalyx composition and thickness were subsequently analyzed. In a first proof-of-concept we utilized the aligned EC monolayers as a tool for cell sheet engineering toward 3-dimensional (3D) modelling of vascular structures. The endothelial barrier and cell-matrix interactions were evaluated by fluorescent staining of tight junctions and focal adhesions.

## 2 Materials and methods

### 2.1 Materials

5-Chlormethylfluoresceindiacetat (CMFDA), anti-chondroitin sulfate antibody (mouse, clone CS-56), anti-vinculin antibody (mouse, Alexa Fluor^®^ 488-conjugated, clone 7F9), anti-ZO-1 antibody (rabbit, polyclonal), DNA-intercalating dye (Hoechst 33342), goat anti-mouse IgG (H+L) (cross-adsorbed, Alexa Fluor^®^ 488-conjugated), goat anti-mouse IgG (H+L) (cross-adsorbed, Alexa Fluor^®^ 647-conjugated), goat anti-mouse IgM (Heavy Chain) (Alexa Fluor^®^ 647-conjugated), goat anti-rabbit IgG (H+L) (Alexa Fluor^®^ 488-conjugated), labelled streptavidin (DyLight™ 488-conjugated), tumor necrosis factor-alpha (TNF-α, human, recombinant protein) and wheat-germ-agglutinin (WGA, Alexa Fluor^®^ 555-conjugated) were purchased from ThermoFisher (Waltham, MA, United States). Cell dissociation buffer (Accutase^®^), Dulbecco’s phosphate-buffered saline (PBS, with and without Ca^2+^ and Mg^2+^), hyaluronic acid binding protein (bovine nasal, biotinylated), octoxynol 9 (Triton™ X-100) and phalloidin (Atto 647N-conjugated) were received from Sigma-Aldrich (St. Louis, MO, United States). Luer adapters for dynamic culture, namely elbow connector [male to female, polypropylene (PP)], luer-lock to barb (male and female, PP), luer-lock to thread (female to 1/4-28 UNF, PP) and panel mount [female to barb with thread (1/4-28 UNF), PP] were ordered from QOSINA (Ronkonkoma, NY, United States). Bovine serum albumin (BSA, fraction V), methanol (>99%), paraformaldehyde (PFA, ROTI^®^Histofix 4%) and polyoxyethylene(20)sorbitan monolaurate (Tween 20^®^) were obtained from Carl Roth (Karlsruhe, Germany). Antibiotic⁄antimycotic solution (gentamicin and amphotericin B), endothelial cell growth medium (VascuLife^®^ VEGF) and human umbilical vein endothelial cells (HUVECs) were purchased from Lifeline^®^ Cell Technology (Frederick, MD, United States) and 6-well plates (tissue culture treated), cell culture flasks (T25, T75, and T175) and rectangular cell culture dishes (quadriPERM^®^) from Sarstedt (Nümbrecht, Germany). Anti-CD31 antibody (mouse, Alexa Fluor™ 488-conjugated, clone JC/70A) and delimiting pen (Dako Pen) were from Agilent Technologies (Santa Clara, CA, United States), hematopoietic cell medium (X-VIVO™ 15, serum-free) and peripheral blood mononuclear cells (PBMCs, human) were from Lonza (Basel, Switzerland). Human fibronectin (FN, lyophilized) was received from Advanced BioMatrix (Carlsbad, CA, United States), polycarbonate (Makroclear^®^) from Arla Plast (Borensberg, Sweden), sterile water for injection from B. Braun (Melsungen, Germany), ethanol (>99%) from Berkel (Ludwigshafen, Germany), mounting medium (ProTaqs^®^ Mount Flour) from Biocyc (Luckenwalde, Germany), rectangular cover glasses (60 **×** 22 mm) from Glaswarenfabrik Karl Hecht (Sondheim vor der Rhön, Germany), 8-well chamber slides (µ-Slide, ibiTreat bottom) from ibidi (Gräfelfing, Germany) and peristaltic pump tubing (Innovaprene^®^ P60) from Innovapure (Shanghai, China). Polystyrene slides (PS, 1.5 mm thickness, transparent) were ordered from Vink König Deutschland (Gilching, Germany) and milled to microscope slide format (76.5 mm, 25.8 mm, 1.5 mm) to fit the slide culture chamber. Fetal bovine serum (FBS, Standard) was from PAN-Biotech (Aidenbach, Germany), endothelial cell growth medium (EGM-2) from PromoCell (Heidelberg, Germany), collagen solution (type I, rat tail) from R & D Systems (Minneapolis, MN, United States), platinum-cured silicon tubing (Tygon^®^ 3350) from Saint-Gobain (Courbevoie, France) and anti-VE-Cadherin antibody (mouse, clone BV9) from Santa Cruz Biotechnology (Dallas, TX, United States). Syringe filters (Minisart^®^, PTFE, 0.2 µm) were obtained from Sartorius (Göttingen, Germany), duplicating silicone (REPLISIL 22 N) from SILCONIC^®^ (Lonsee, Germany) and anti-heparan sulfate antibody (mouse, clone 8.S.087) from US Biological (Salem, MA, United States).

### 2.2 Ethics statement

Human tissue was obtained after approval by the local Ethics Committee (Hannover Medical School, Ethical approval No: 844-2010) and following the donor’s written informed consent, or in the case of newborns, following informed consent of the parents.

### 2.3 Cell culture

HUVECs (passage 2–6) were cultured in VascuLife VEGF medium supplemented with 30 µg ml^−1^ gentamicin, 15 ng ml^−1^ amphotericin B and 2% FBS. iPSCs (MHHi001-A) were maintained under standard culture conditions (Haase et al., 2017) and differentiated toward iPSC-ECs according to Olmer et al. (2018). iPSC-ECs (passage 2–4) were maintained in EGM-2 medium supplemented with 30 µg ml^−1^ gentamicin, 15 ng ml^−1^ amphotericin B and 2% FBS. All cell culture substrates for iPSC-ECs were coated with 2.5 µg cm^−2^ human fibronectin in PBS^+^ for 30 min under standard conditions (5% CO_2_, 37 °C) and washed once with sterile water prior to cell seeding. ECs were sub-cultured using Accutase^®^ cell dissociation reagent once reaching a confluency of 70–80%.

Unless otherwise mentioned, cell culture experiments were performed on thermoresponsive poly (glycidyl methyl ether-*co*-ethyl glycidyl ether)-coated (PGE-coated) polystyrene slides prepared as described before (Stöbener et al., 2018; Stöbener and Weinhart, 2020). After coating, slides were transferred to rectangular cell culture plates, disinfected with 70% ethanol for 10 min at room temperature (RT) and washed twice with cold (4 °C) PBS^+^. Sterilized cast silicone cell separators with a seeding area of 3 cm^2^ were placed onto slides and cells were seeded with a density of 42,500 cells cm^−2^ in 300 µl cm^−2^ VascuLife VEGF medium containing 2% FBS. After 24 h the cell culture medium was replaced with medium containing 10% FBS and the cells were kept for two more days under standard conditions to reach confluency. Cells were now either maintained under static conditions or transferred to dynamic culture for additional 96 h, which is referred to as “static 96 h” and “dynamic 96 h” throughout the manuscript.

### 2.4 Dynamic culture: Setup and flow characterization

**FIGURE 1:**
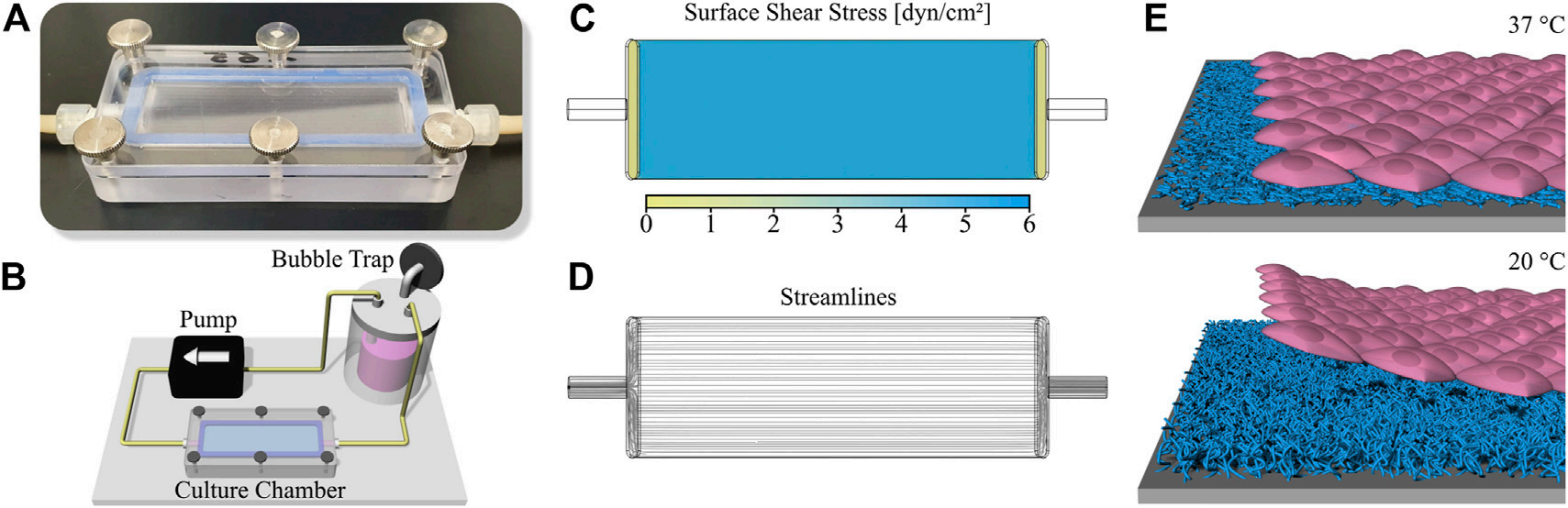
Setup for dynamic cell culture and cell sheet fabrication. **(A)** Photography of the slide culture chamber (channel height 150 µm) and **(B)** illustration of the circuit for dynamic cell culture including medium reservoir/bubble trap, peristaltic pump and culture chamber. Characterization of the comprised flow channel *via* Computational Fluid Dynamics (CFD) simulation showing **(C)** surface shear stress and **(D)** flow streamlines. **(E)** Schematic illustration of cell sheet fabrication using a thermoresponsive PGE-coating on polystyrene (PS). The polymer coating is cell adhesive at a temperature of 37 °C while changing to a cell repellent state at 20 °C, therefore initiating the enzyme-free detachment of confluent cell sheets after the temperature switch.

Cells were cultured under fluid flow conditions in parallel plate flow chambers as described before (Lindner et al., 2021). In brief, culture chambers fabricated from polycarbonate which can accommodate a standard microscopy slide are used to apply a homogenous shear stress onto the cell surface (Figure 1A). The chamber is included into a simple circuit comprising a bubble trap/medium reservoir and a peristaltic pump is used to generate a constant fluid flow (Figure 1B). With a channel height of 0.15 mm and a volume flow rate of 3.4 ml min^−1^, the applied shear stress is approximately 6.6 dyn cm^−2^ (Lindner et al., 2021). The final pump speed was gradually increased in three steps with an interval of 45 min to initially allow cells to adapt to the shear stress. CFD simulations were performed as previously published (Lindner et al., 2021), confirming laminar flow and homogenous surface shear stress (Figures 1C,D).

### 2.5 Directionality analysis

Phase-contrast images of ECs after static and dynamic culture were acquired using an inverted microscope (Axio Vert.A1, Carl Zeiss, Jena, Germany) equipped with a monochrome camera (Axiocam MRm, Carl Zeiss). Subsequently, the directionality of cells was analyzed either qualitatively by adding an angle-dependent color to cell borders or quantitatively by determining the main orientation angle of individual cells. Both analyses were carried out using *Fiji*, an *ImageJ* distribution for scientific image analysis (Schindelin et al., 2012). The processing is described in detail in Supplementary Section 2.

### 2.6 Immunofluorescent staining

For immunofluorescent staining, cells were rinsed with PBS^+^ after static and dynamic culture and fixed in cold methanol (-20 °C) or 4% PFA for 10 min at 4 °C or RT, respectively. PFA-fixed samples were permeabilized with 0.1% (v/v) Triton-X 100 in PBS^+^ for 5 min at RT and both types of samples were blocked with 5% BSA (w/v) in PBS^+^ containing 0.05% (v/v) Tween (PBST) for 90 min at RT. The endothelial marker CD31, glycocalyx components heparan sulfate (HS), chondroitin sulfate (CS) and hyaluronic acid (HA), as well as proteins of tight junctions (zonula occludens protein 1 (ZO-1) and vascular endothelial cadherin (VE-cadherin)) were detected *via* indirect immunofluorescence, whereas the actin cytoskeleton and focal adhesions were stained directly *via* phalloidin and labeled vinculin antibodies, respectively. Staining conditions are listed in Table 1. After staining, the samples were mounted with mounting medium and glass coverslips. Confocal microscopy was performed using Zeiss LSM800 equipped with an Airyscan detector (Carl Zeiss).

**TABLE 1:** Conditions for immunofluorescence staining.

Primary antibody/protein	Secondary antibody/protein
Mouse-α-CD31^a^, IgG1, 1:100	Goat-α-mouse IgG (H+L) Alexa Fluor^®^ 488, 1:500
Mouse-α-chondroitin sulfate^a^, IgM, 1:100	Goat-α-mouse IgM Alexa Fluor^®^ 647, 1:500
Mouse-α-heparan sulfate^b^, IgM, 1:100	Goat-α-mouse IgM Alexa Fluor^®^ 647, 1:500
Hyaluronic acid binding protein^b^, biotinylated, (5 µg ml^−1^)	Streptavidin DyLight™ 488, 1:250
Mouse-α-vinculin^a^, Alexa Fluor® 488, 1:50^d^	-
Mouse-α-VE-cadherin^b,c^, IgG2a, 1:100	Goat-α-mouse IgM Alexa Fluor^®^ 647, 1:500
Rabbit-α-ZO 1^b,c^, polyclonal, 1:250	Goat-α-rabbit IgG (H+L) Alexa Fluor^®^ 488, 1:500

Samples were fixed with ^a^PFA or ^b^methanol, ^c^indicates co-staining, ^d^counter staining using Phalloidin, Atto647N, 1:250

### 2.7 Glycocalyx thickness determination

To measure the glycocalyx thickness after fixation and staining, confocal Z-stacks of CS stained samples were acquired under Airyscan super-resolution mode, processed with *ZenBlue* software and analyzed with *ImageJ* as described previously (Tiemeier et al., 2019). In brief, Z-stacks were resliced to yield orthogonal projections. Subsequently, intensity profiles (Z-direction) for nucleus and CS staining were plotted and a gaussian fit was added. The thickness was calculated as the difference between apical full width half maximum of nucleus to CS signal. A minimum of seven spots per cell and four cells per cell type and culture condition were analyzed. Airyscan processing strength was kept constant for all images to allow comparison between single conditions and imaging was done considering optical limitations.

### 2.8 Peripheral blood mononuclear cell adhesion assay

#### 2.8.1 Labelling

The adhesion of peripheral blood mononuclear cells (PBMCs) to ECs after static and dynamic conditions was quantified to assess glycocalyx functionality. For every experiment, one vial containing 10 **×** 10^6^ PBMCs was thawed in a water bath (37 °C), transferred to 9 ml prewarmed X-Vivo medium and gently mixed *via* pipetting. Cells were centrifuged for 10 min at 300 **×** g and the supernatant was aspirated. Cells were then resuspended in 5 ml prewarmed medium with CMFDA (10 µM) and labelled for 5–10 min in a cell culture incubator. After two repeated centrifugation (10’, 300 **×** g) and resuspensions steps, cells were resuspended in 1 ml medium, counted and further diluted to a final concentration of 5 **×** 10^5^ cells ml^−1^.

#### 2.8.2 Incubation and data acquisition

PS-slides with ECs were carefully rinsed once with X-Vivo medium and 300 µl cm^−2^ of the PBMC-suspension were added. After incubation for 60 min in an incubator, slides were washed with prewarmed PBS^+^ and fixed with PFA for 10 min at RT. The samples were finally imaged *via* confocal microscopy and data were analyzed using *ImageJ*. Details about the assay validation, image processing and analysis can be found in Supplementary Section 4.

### 2.9 Cell sheet engineering

To harvest confluent HUVEC or iPSC-EC sheets, slides with adherent cells were transferred to RT-PBS^−^ after application of different culture conditions and incubated for 10–15 min. Cell sheet detachment started spontaneously at the edges of the cell layer and was monitored *via* phase contrast microscopy. To fully detach sheets from the substrate, gentle flushing with a small-volume pipette was necessary.

### 2.10 Cell sheet rolling

In a preliminary experiment, dynamically cultured iPSC-ECs were wrapped around a 3D-printed tubular scaffold as recently reported for statically cultured HUVECs (Elomaa et al., 2022). In brief, a custom-made rolling device (OSPIN, Berlin, Germany) was used to transfer the EC sheets onto a 3D printed tubular scaffold while detaching from the substrate. Subsequently, the tubular construct was transferred to an 8-well slide and embedded into a collagen hydrogel. For this, 500 µl acidic rat tail collagen solution (5 mg ml^−1^) were mixed with 110 µl 0.1 M NaOH, 100 µl 10× PBS and 290 µl cell culture grade water. The solution was added to the well containing the tube and solidified for 30 min at 37 °C. Then, the cytoskeleton and nuclei were stained with phalloidin (1:250) and Hoechst 33342 (1:1000) in PBS^−^ for 3 h at RT, respectively. After five washing steps for 30 min each with PBS^−^, the samples were imaged *via* confocal microscopy to estimate coverage of the scaffold and cell directionality after the rolling process.

### 2.11 Statistics

Statistical analysis was performed with the software package *Origin Pro*. In case of normally distributed data, comparison was done using two-way ANOVA followed by Bonferroni post hoc test, whereas non-normally distributed data was compared *via* Mann-Whitney Test. Statistical significance is indicated with *p*-values * < 0.05 and *** < 0.0005.

## 3 Results

### 3.1 Cellular orientation of HUVECs and iPSC-ECs in flow direction

To comparatively analyze the effect of shear stress applied to the apical surface of HUVEC or iPSC-EC monolayers on cellular orientation as well as glycocalyx composition and thickness a previously established reusable slide cultivation chamber was deployed for the culture of cells under flow on conventional microscopy slides (Figure 1A) (Lindner et al., 2021). This chamber can be implemented into a simple circuit further comprising a peristaltic pump and a medium reservoir simultaneously working as a bubble trap (Figure 1B). Operation of the circuit inside a customary cell culture incubator allows control of temperature and pH value. The setup enables the application of homogenous surface shear stress and laminar flow of the medium to adherent cells (Figures 1C,D). To facilitate the generation of confluent monolayers comprising a flow-induced cellular orientation for potential tissue engineering applications the ECs were cultured on thermoresponsive surfaces. These surfaces reversibly respond to temperature and are cell adhesive at a temperature of 37 °C but become cell repellent after a temperature switch to 20 °C which initiates the enzyme-free detachment of single cells or confluent cell sheets (Figure 1E). To prepare such substrates, untreated polystyrene (PS) slides were modified with a _~_ 5 nm thin, thermoresponsive poly(glycidyl ether) (PGE) brush coating (Stöbener et al., 2018; Stöbener and Weinhart, 2020).

It is well known, that ECs undergo elongation as well as alignment in the direction of fluid flow after application of shear stress to the cell surface (Thoumine et al., 1995; Steward et al., 2015). To obtain information about intensities of shear stress and time periods needed to induce flow-alignment of HUVECs in comparison to iPSC-ECs in our *in vitro* setup, a preliminary analysis was performed on conventional tissue culture PS (TCPS) culture substrates applying physiological shear stresses between 0 and 10 dyn cm^−2^ to monolayers. Results indicate a dependency of HUVEC alignment on both time and shear force (Supplementary Figure S1). While there was no directionality of both cell types after static culture, HUVECs showed an alignment after 48 h for shear stresses of 6.6–10 dyn cm^−2^, after 72 h for 3.3–5 dyn cm^−2^ and after 96 h for 1.7–2.5 dyn cm^−2^. iPSC-ECs on the other hand were already fully aligned after 24 h of culture with a shear force as little as 1.7 dyn cm^−2^. For the highest shear stress of 10 dyn cm^−2^ we observed a reduction of confluency of the HUVEC monolayer at 96 h compared to earlier time points (Supplementary Figure S1). To allow a comparison between the effects of continuous fluid flow on both cell types we decided to stick to a shear stress of 6.6 dyn cm^−2^ and a time span of 96 h for further experiments to enable fast alignment of cells without causing defects in the monolayer during the 96 h culture time. This flow condition applied to cells cultured on thermoresponsive PGE-modified PS substrates will from now on be referred to as “dynamic culture”.

**FIGURE 2:**
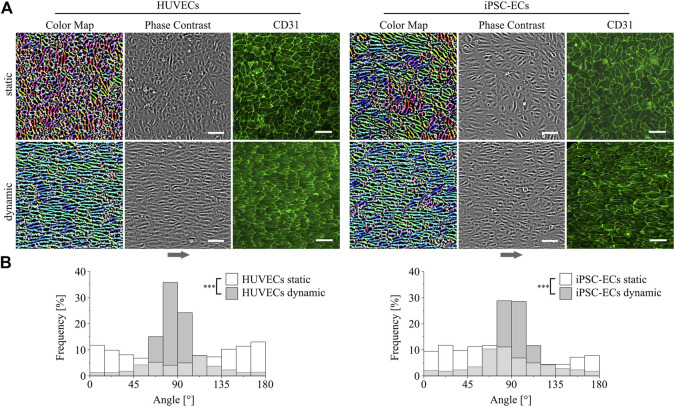
Directionality and differentiation analysis of HUVECs and iPSC-ECs on PGE-coated PS after culture with or without flow. **(A)** Qualitative cellular alignment after static and dynamic culture of HUVECs and iPSC-ECs (96 h), shown by phase contrast and corresponding color-coded images indicating cellular orientation by uniformity of coloration. Differentiation status was confirmed by confocal imaging of CD31 (green) as endothelial marker (independent sample) (Arrow indicates flow direction. Scale bars: 100 µm). **(B)** Quantitative orientation analyses determined from brightfield images after static and dynamic culture of HUVECs and iPSC-ECs (96 h) shown as histograms. Significant differences were found between each dynamic and the respective static condition for both cell types, respectively (*n* = 3, Mann-Whitney Test, *** *p* < 0.0005).

*In vitro* ECs generally show a cobblestone-like phenotype when cultured as a monolayer without fluid flow (Baudin et al., 2007; Adams et al., 2013). This was also found for HUVECs as well as iPSC-ECs cultured under static conditions on PGE-coated surfaces as it can be seen in Figure 2A from phase contrast and color-coded images. The latter were obtained *via* image processing as described in the Supplementary material (Supplementary Figure S2). Regarding the coloration of the image a higher uniformity of the color indicates a higher degree of orientation of the cells. This was observed for dynamic culture for both types of ECs which are aligned in the direction of the fluid flow (Figure 2A). Quantitative analysis of cellular directionality (Supplementary Figure S3) validated these results as shown in distribution plots in Figure 2B. For both HUVECs and iPSC-ECs there was a significant difference in main orientation between a more or less homogeneous distribution of cellular directionality after static and a narrower distribution after dynamic culture (Figure 2B). A CD31-staining revealed preservation of the EC status of both cell types under static as well as dynamic conditions on thermoresponsive polymer coatings (Figure 2A).

### 3.2 Effects of shear stress on HUVEC and iPSC-EC glycocalyx composition and thickness

**FIGURE 3:**
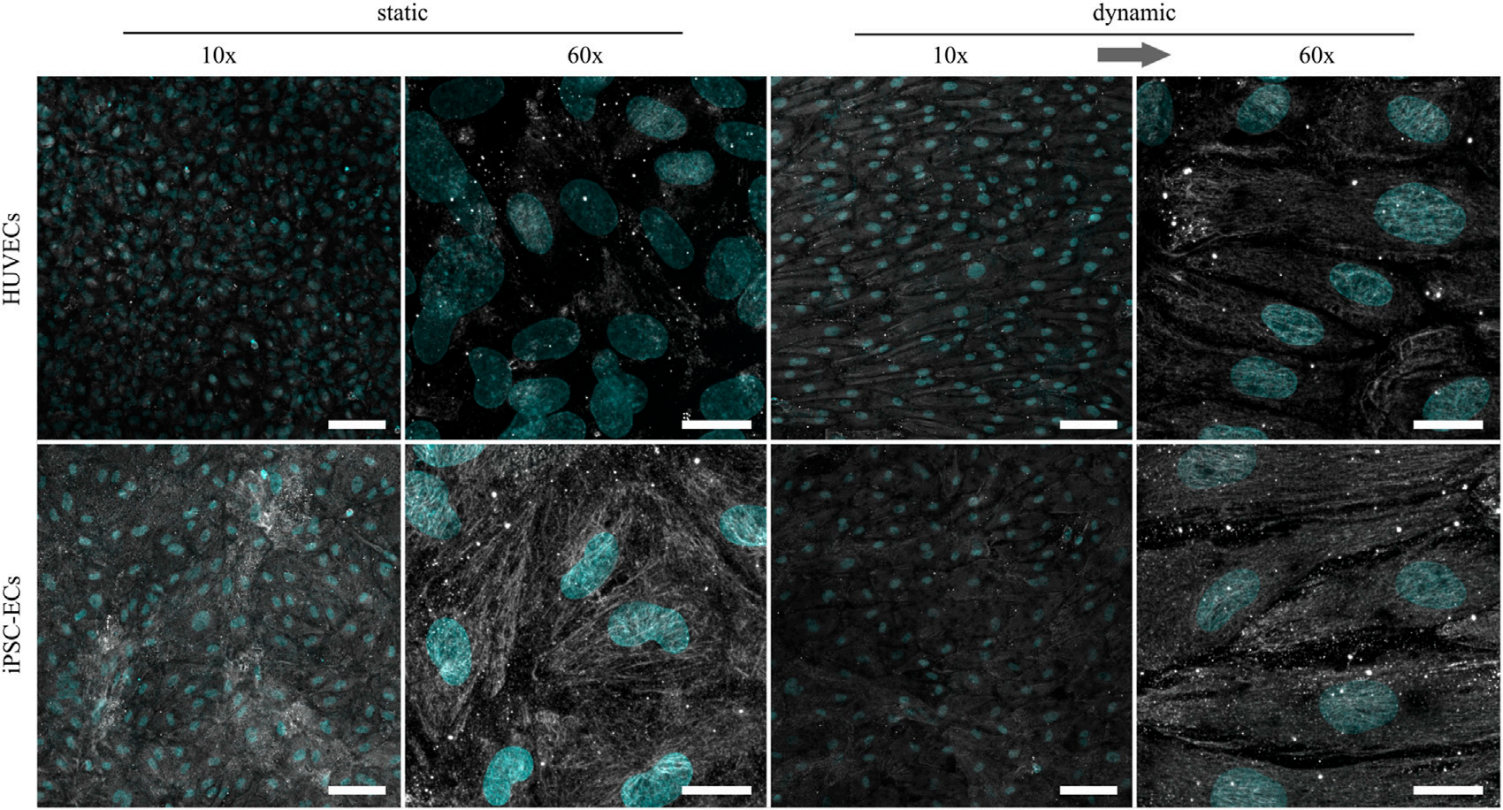
Representative confocal immunofluorescence images of a chondroitin sulfate staining (grey) of HUVECs and iPSC-ECs after 96 h of static and dynamic culture. Nuclei were counterstained with Hoechst 33342 (cyan). (Arrow indicates flow direction. Scale bars: 10× 100 µm, 60× 20 µm).

**FIGURE 4:**
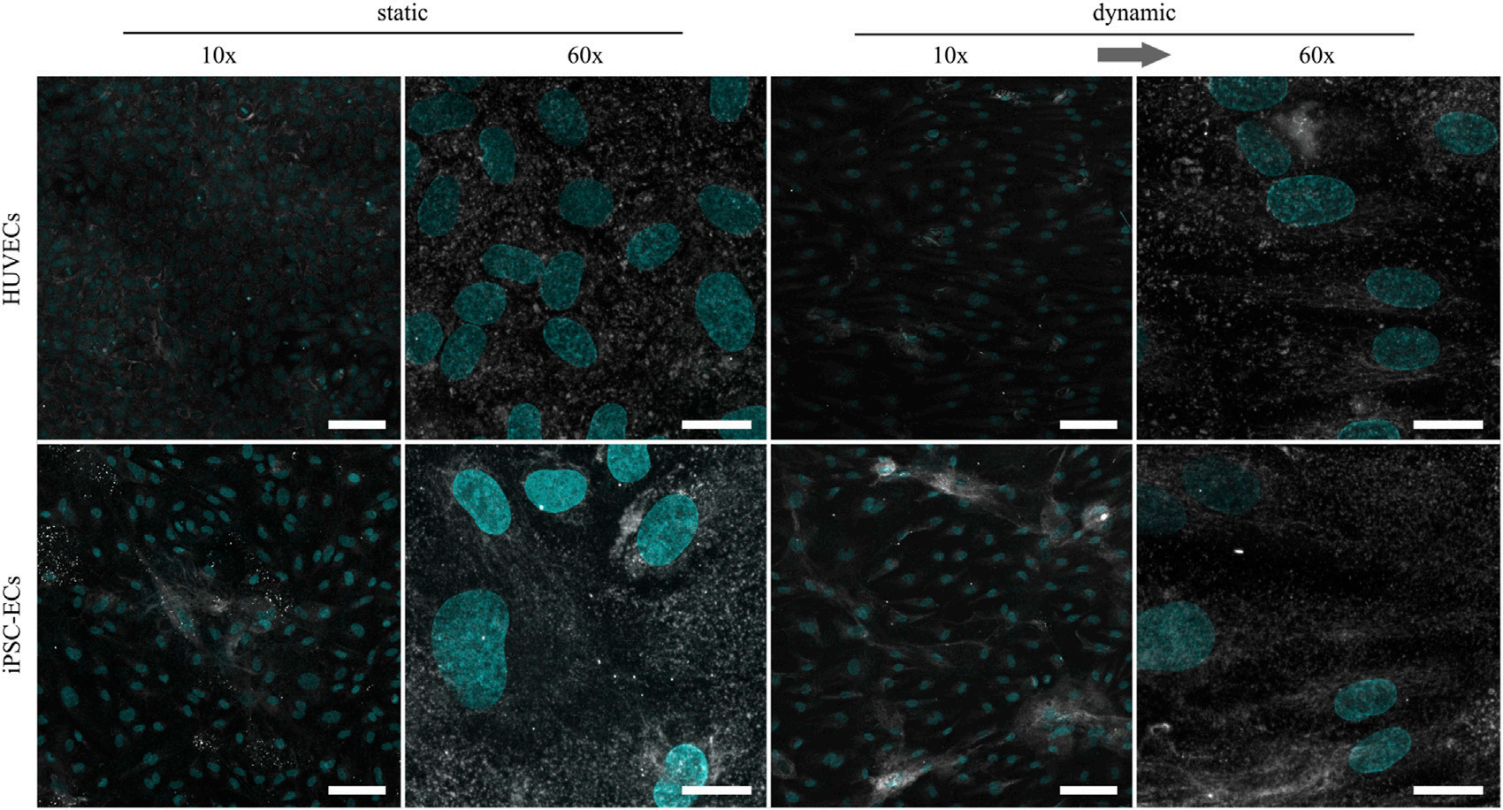
Representative confocal immunofluorescence images of a hyaluronic acid staining (grey) of HUVECs and iPSC-ECs after 96 h of static and dynamic culture. Nuclei were counterstained with Hoechst 33342 (cyan). (Arrow indicates flow direction. Scale bars: 10× 100 µm, 60× 20 µm).

**FIGURE 5:**
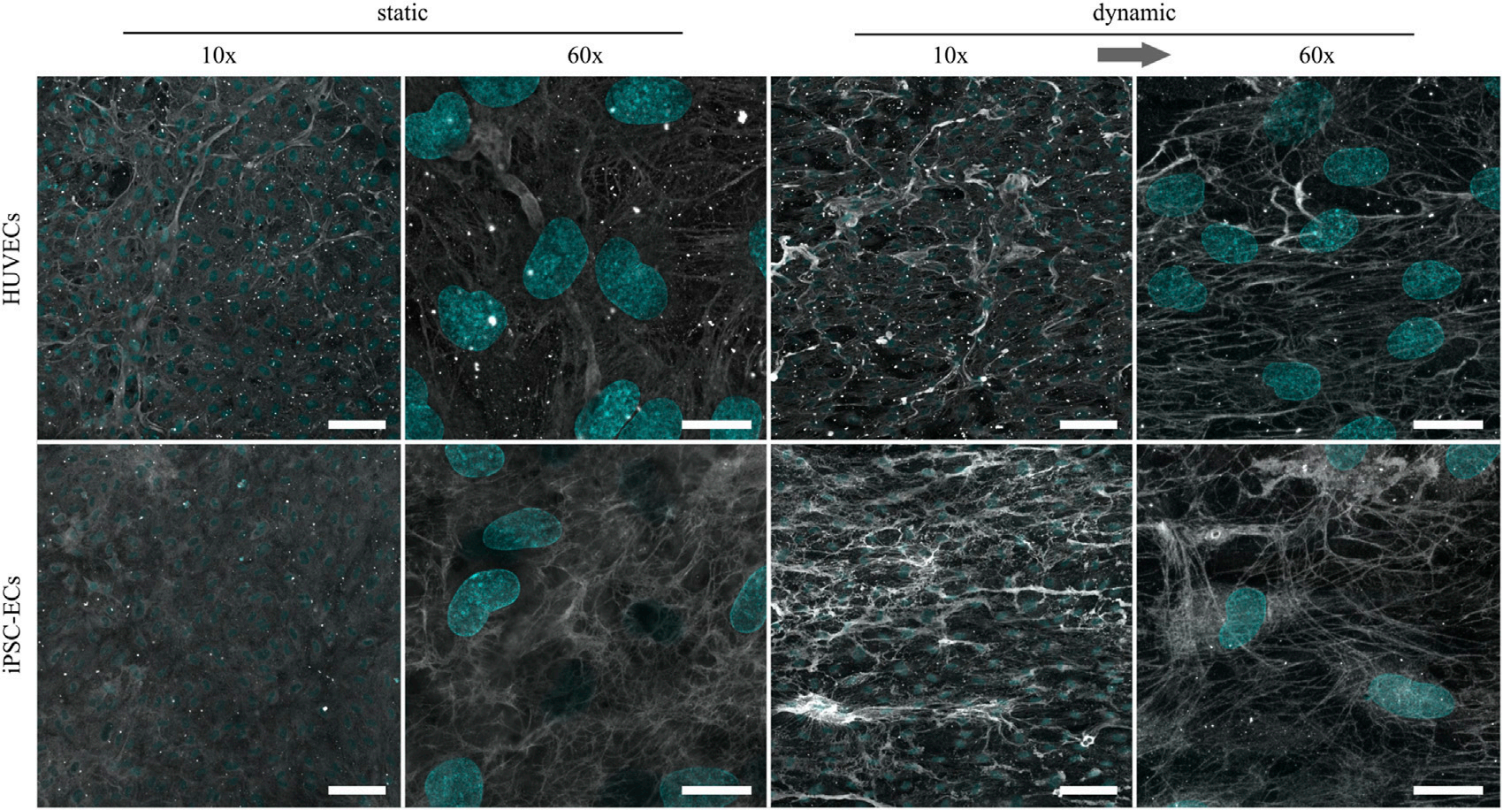
Representative confocal immunofluorescence images of a heparan sulfate staining (grey) of HUVECs and iPSC-ECs after 96 h of static and dynamic culture. Nuclei were counterstained with Hoechst 33342 (cyan). (Arrow indicates flow direction. Scale bars: 10× 100 µm, 60× 20 µm).

As previously shown, the glycocalyx composition as well as thickness in ECs changes depending on the applied shear stress (Gouverneur et al., 2006a; Potter and Damiano, 2008). To further investigate possible changes in HUVEC and iPSC-EC glycocalyx composition, staining of the three main glycosaminoglycans (GAGs), namely chondroitin sulfate (CS), hyaluronic acid (HA) and heparan sulfate (HS), was performed after static and dynamic culture (Figures 3–5). Under static conditions the amount of CS on the cell surface turned out to be distinctly higher in iPSC-ECs compared to HUVECs, while this difference dissipated after dynamic culture particularly due to an increase in CS on the surface of HUVECs. Considering structural features, the CS formed a dense mesh-like pattern uniformly covering the cell surfaces, that also aligned with the fluid flow after dynamic culture (Figure 3, 60× magnification).

Regarding the amount of HA, there were no marked differences between HUVECs and iPSC-ECs after static culture (Figure 4). Structurally the HA seemed to be more condensed in some spots, especially in the case of iPSC-ECs, and not as homogeneously distributed as CS described before. For iPSC-ECs the amount of HA comprised in the clusters increased after dynamic culture, while this trend was not observed to this extend in the case of HUVECs cultured under flow (Figure 4, 10× magnification). Evaluation of the images acquired at a magnification of 60× revealed a relatively uniform HA distribution with no mesh or fiber formation (Figure 4, 60× magnification).

Being the most abundant GAG component of the glycocalyx and also known as mechanosensor (Oohira et al., 1983; Ihrcke et al., 1993; Ebong et al., 2014), HS revealed the most expressive changes due to culture conditions. While in both culture conditions there was no drastic difference in the amount of HS on HUVECs compared to iPSC-ECs, there was a clear increase in the HS amount after dynamic culture for both cell types compared to static culture (Figure 5). Regarding the phenotype, HS formed a dense outstretched mesh across the cellular monolayer with generation of some thicker fibers in the case of HUVECs after static culture. Application of shear stress induced the formation of thicker HS fibers which were still arranged as a dense network but additionally aligned with the flow direction (Figure 5).

**FIGURE 6:**
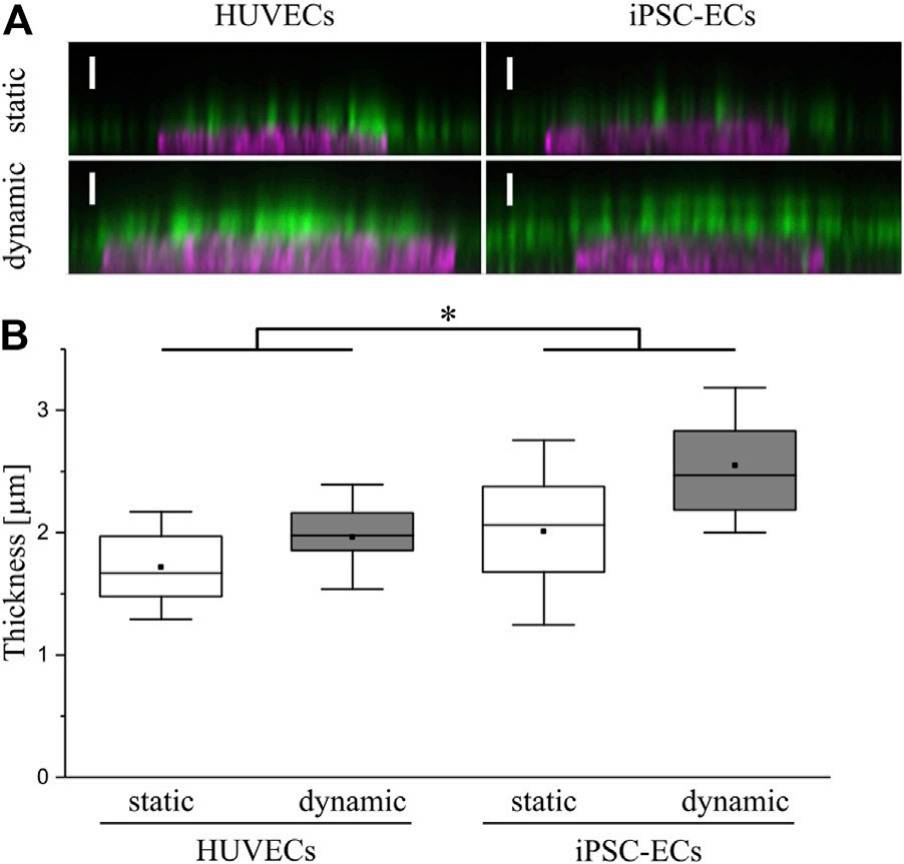
Determination of glycocalyx thickness on HUVECs and iPSC-ECs after 96 h of static and dynamic culture. **(A)** Representative confocal images of chondroitin sulfate (green) and nuclear staining (magenta) shown as orthogonal projection (Scale bars: 2.5 µm) to determine **(B)** the glycocalyx layer thickness. Data are presented as box plots including mean, median and 90% confidence interval. Significant differences were found between overall HUVEC vs iPSC-EC samples as well as between overall static vs dynamic samples deployed as the main factors (*n* = 4, two-way ANOVA: main factors, * *p* < 0.05). Comparing single conditions the only significant difference was found between HUVEC static vs iPSC-EC dynamic (*p* < 0.006).

For analysis of the influence of surface shear stress on glycocalyx thickness confocal images of the homogeneously distributed CS staining with Hoechst counterstaining were employed (Figure 6A) as previously performed elsewhere (Tiemeier et al., 2019). To estimate the glycocalyx thickness the distance between the half-maximal signal of the nuclear staining representing the apical cell surface and the half-maximal signal of the luminal end of the CS staining was determined for both cell types and culture conditions. Comparing layer thicknesses on the surface of HUVECs and iPSC-ECs, there was a significantly thicker glycocalyx observed for the iPSC-ECs (Figure 6B). Additionally, the measurement revealed a significant difference between static and dynamic culture conditions with a higher glycocalyx thickness for dynamic culture, ranging between 2.0 ± 0.3 µm for HUVECs and around 2.5 ± 0.5 µm for iPSC-ECs (Figure 6B). As this analysis was conducted after fixation of cells, which potentially causes a collapse of the glycocalyx and thereby underestimates the thickness, we additionally performed a live-cell staining using wheat germ agglutinin (WGA) to verify the obtained results (Supplementary Figures S4, S5). Lacking the fixation, it was necessary to switch the culture substrate from the thermoresponsive polymer coating toward a non-responsive TCPS surface, thereby preventing unintended, spontaneous detachment of cells during image acquisition. First a lectin staining of cells cultured under static conditions on both substrates was conducted for HUVECs and iPSC-ECs, respectively (Supplementary Figure S4A), demonstrating no influence of the culture substrate on glycocalyx expression (Supplementary Figure S4B). Imaging of native WGA staining together with cytosolically located CMF-DA showed a glycocalyx thickness of around 1.5–2.5 µm after static culture of HUVECs and iPSC-ECs and thereby verified the results obtained from CS staining after fixation (Supplementary Figure S5).

### 3.3 Effects of shear stress on glycocalyx functionality

To evaluate the functionality of the glycocalyx formed on HUVECs and iPSC-ECs under static as well as dynamic culture conditions a peripheral blood mononuclear cell (PBMC) adhesion assay was performed. In general, PBMCs are blood cells exhibiting a single round nucleus, for, example, lymphocytes, monocytes, natural killer cells and dendritic cells. As integral parts of the immune system these cell types are able to interact with the luminal endothelium, for example, in the case of inflammation, undergoing the well-known steps of capture, rolling, adhesion and transmigration through the endothelial layer. This complex mechanism is controlled by a myriad of factors and processes, one being the decline in glycocalyx thickness by macromolecular shedding granting access to shorter membrane bound glycoproteins like selectins and adhesion molecules (intercellular adhesion molecules (ICAMs), vascular cell adhesion molecules (VCAMs)) on the EC surface (Kalucka et al., 2017). Therefore, the PBMC adhesion assay can be used as a simple method to evaluate glycocalyx functionality *in vitro* giving a lower count of adhering PBMCs with higher glycocalyx integrity and thickness.

**FIGURE 7:**
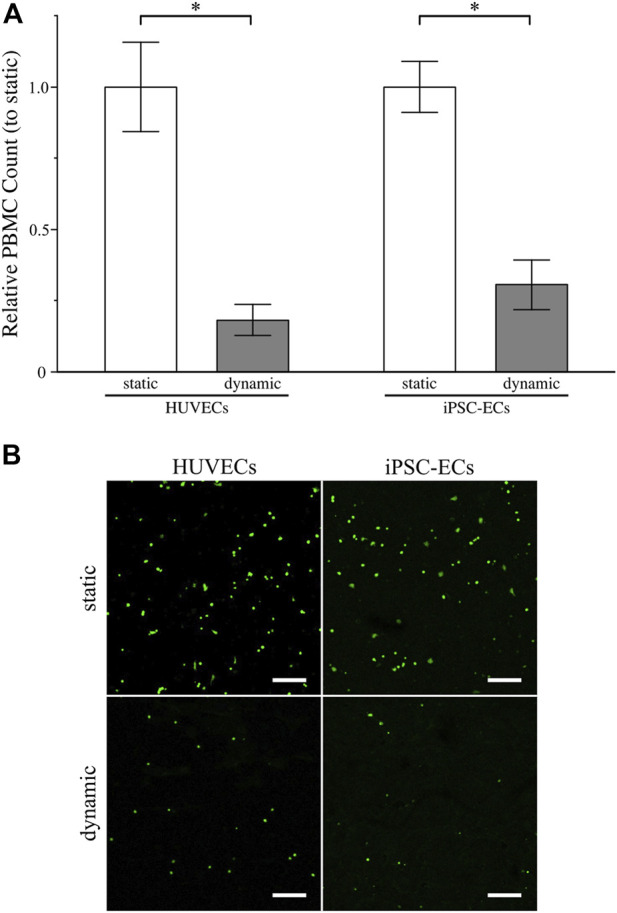
Assessment of glycocalyx functionality of HUVECs and iPSC-ECs after 96 h of static and dynamic culture *via* PBMC adhesion assay. **(A)** Relative PBMC count determined from confocal images (for details on image processing refer to Supplemental Figure S6A). Data are presented as mean ± SEM with respect to “static” (*n* = 4, Mann-Whitney Test, * *p* < 0.05) **(B)** Representative unprocessed confocal images of adherent PBMCs labelled with CMFDA on the surface of confluent HUVEC and iPSC-EC monolayers. (Scale Bars: 200 µm).

For establishment of the assay, we performed PBMC adhesion using HUVEC monolayers with different concentrations of CMFDA-labelled PBMCs as well as potent positive controls (Supplementary Figure S6). The adhered PBMC counts increased with increasing cell numbers used in the assay (Supplementary Figure S6C). Additionally, mechanical and especially cytokine-induced glycocalyx shedding led to a drastic increase in PBMC adhesion, thereby proofing the functionality of the assay (Supplementary Figure S6B,C). When analyzing the influence of culture conditions on PBMC adhesion to HUVECs or iPSC-ECs we found a significant decrease in PBMC adhesion after dynamic culture for both cell types compared to static culture (Figures 7A,B), arguing for a better glycocalyx functionality/integrity after application of shear stress. This goes in line with the finding of higher amounts of HS on the EC surfaces and increased glycocalyx thicknesses after dynamic culture. Comparison of leucocyte adhesion to HUVECs and iPSC-ECs revealed no significant difference regarding the cell type (Supplementary Figure S7).

### 3.4 Detachment of aligned HUVEC and iPSC-EC sheets from thermoresponsive polymer coatings

**FIGURE 8:**
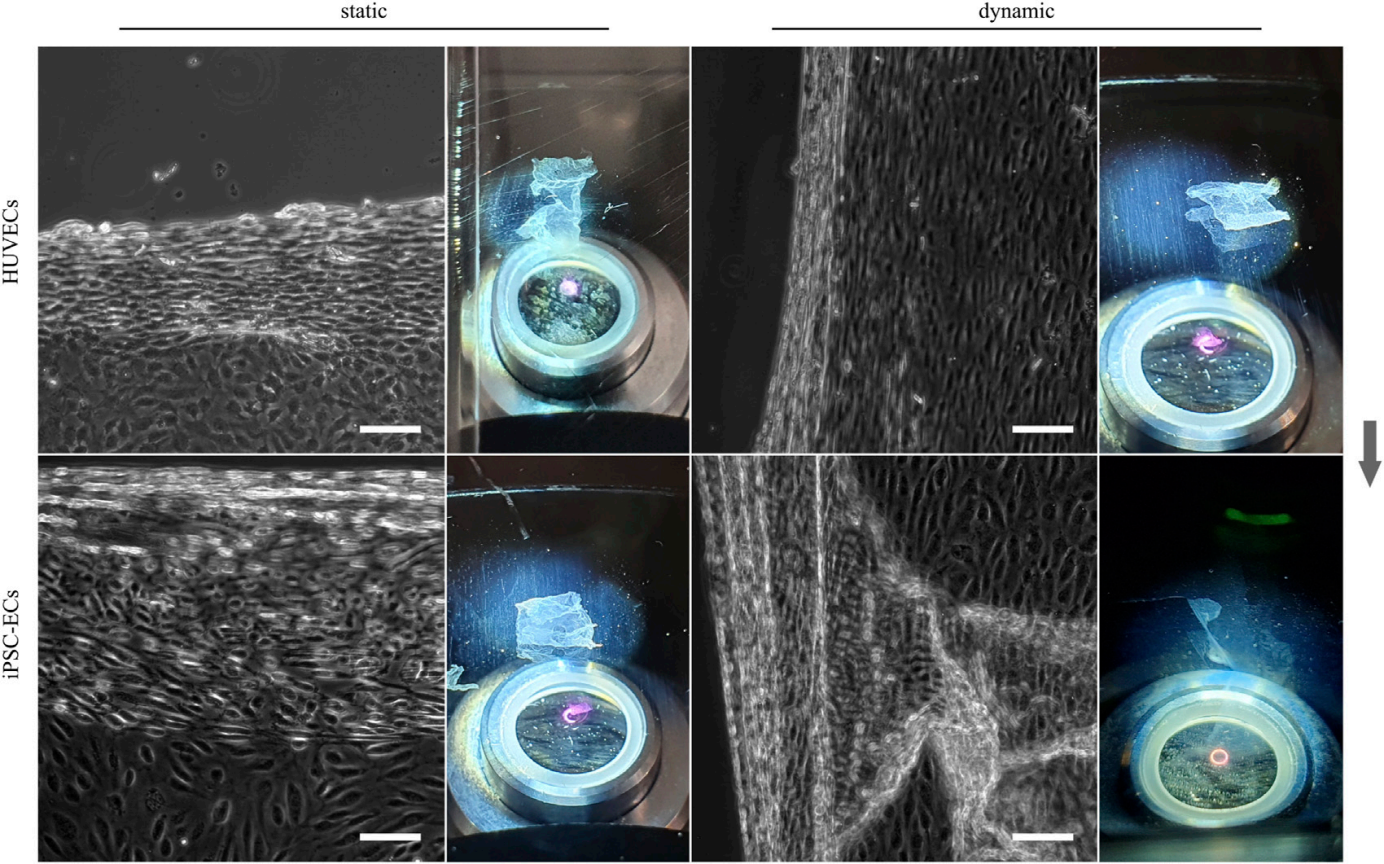
Enzyme-free harvest of HUVEC and iPSC-EC cell sheets from PGE-coated PS substrates triggered by a short temperature switch after static and dynamic culture for 96 h. Detachment of cell sheets is shown for each condition by a representative phase contrast image as well as a photograph (Arrow indicates flow direction. Scale bars: 100 µm, *n* = 3).

In addition to the analysis of cellular alignment under flow conditions and the study of glycocalyx alterations due to shear stress, one objective of this study was the generation of polarized, confluent HUVEC and iPSC-EC cell sheets, preferably with cells in an aligned state after dynamic culture for tissue engineering applications. A first attempt to detach confluent cellular monolayers from thermoresponsive PGE-coated surfaces after 48 h, which is the time point sufficient for cellular alignment at the applied shear stress, resulted in singularization or partial sheet detachment of HUVECs and iPSC-ECs after both static or dynamic culture (Supplementary Figure S8). Extending the culture time to 72 h improved the integrity of detached sheets, yet it was still not possible to harvest fully intact cell sheets from any of the conditions (data not shown). For both HUVECs and iPSC-ECs, cell sheet fabrication was possible after 96 h of culture without flow as shown by phase contrast images and photographs in Figure 8. Intact aligned HUVEC cell sheets also detached after 96 h of shear application, whereas the iPSC-EC layer indeed separated from the thermoresponsive surface but lost its integrity resulting in a disrupted sheet (Figure 8). It was evident that aligned cell sheets tended to detach first from the sides parallel to the applied fluid flow.

**FIGURE 9:**
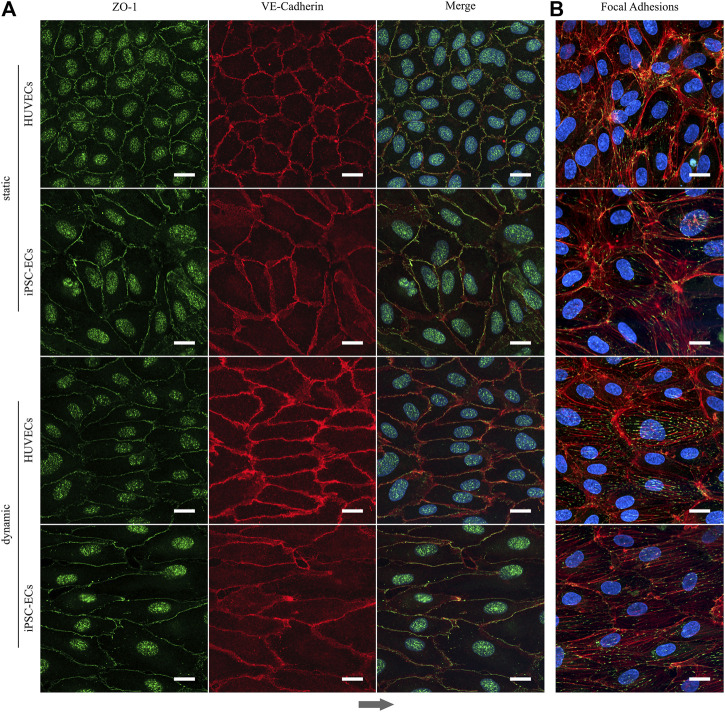
Formation of tight junctions and appearance of focal adhesion after static and dynamic culture of HUVECs and iPSC-ECs for 96 h. **(A)** Representative confocal images of ZO-1 (green) and VE-Cadherin staining (red). Nuclei were counterstained with Hoechst 33342 (blue). **(B)** Confocal images of phalloidin (red) and vinculin staining (green) representing the focal adhesions. Nuclei were counterstained with Hoechst 33342 (blue). (Arrow indicates flow direction. Scale bars: 20 µm).

Searching for a reason for the differential behavior of aligned iPSC-ECs regarding the cell sheet detachment, we decided to conduct staining verifying the expression of essential components that ensure the integrity of endothelial layers. After 96 h of both static and dynamic culture ZO-1 as a protein associated with tight junctions is present in HUVECs as well as iPSC-ECs (Figure 9A). Same is true for VE-cadherin which is an indicator for EC junction integrity. Both proteins co-localize at the cell-cell interface and for cells cultured under dynamic conditions the alignment of cells along the flow is evident in the microscopic images (Figure 9A). As there were no differences in occurrence of cell-cell contacts we additionally stained the cells for F-actin and vinculin investigating the formation of stress fibers and focal adhesions. Indeed, the staining revealed an increased formation of stress fibers in the direction of flow in aligned cells after application of shear stress with a pronounced formation of focal adhesions (Figure 9B, Supplementary Figure S9). Since this was determined for both HUVECs and iPSC-ECs this finding does not explain the difficulties in intact, self-detached cell sheet fabrication observed for iPSC-ECs.

**FIGURE 10:**
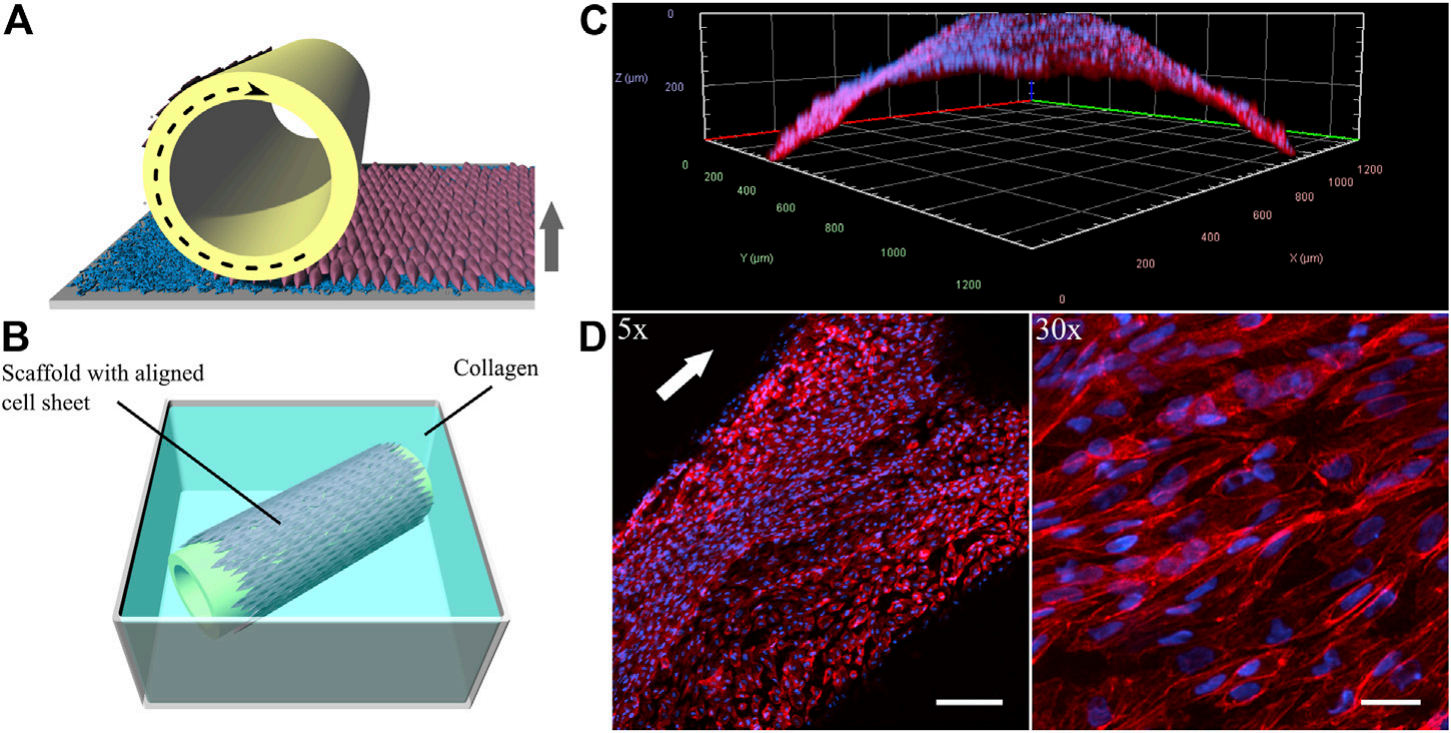
Generation of vessel-like constructs by transferring a detaching aligned endothelial sheet from a PGE-coated thermoresponsive substrate onto a tubular 3D-printed scaffold. **(A)** Illustration of wrapping process and **(B)** rolled sheet on scaffold embedded in a collagen gel. **(C)** 3-dimensional reconstruction and **(D)** Z-projection of confocal stacks of the phalloidin-stained (red) iPSC-EC sheet at different magnifications. Nuclei were counterstained with Hoechst 33342 (blue). Cell sheet was detached and rolled after 96 h of dynamic culture. (Arrows indicate flow direction. Scale bars: 5× 200 µm, 30× 30 µm).

With vascular tissue engineering applications in mind, which largely benefit from a polarized, aligned EC sheet, we tackled the issue of the incomplete self-detachment of the iPSC-EC sheet by immediately transferring it onto a 3D printed tubular scaffold during the detachment process. This was accomplished by a recently developed procedure in our group using a custom-made rolling device (Elomaa et al., 2022) as illustrated in Figure 10A. The biofabricated vessel mimic made from a dynamically cultured iPSC-EC sheet in this preliminary proof-of-concept study was embedded into a collagen hydrogel (Figure 10B). The coverage of the tubular scaffold is shown by a 3D-reconstruction indicating the curvature of the 3D-printed structure (Figure 10C) as well as by a Z-projection of confocal stacks (Figure 10D). The latter not only proves the continuous coverage of the rod but also shows the intact alignment of the cells after detachment and rolling of the sheet.

## 4 Discussion

In this study we investigated the influence of physiological shear stress on cellular alignment and glycocalyx formation of HUVECs compared to iPSC-ECs. In order to apply homogenous laminar flow to the cellular monolayers our recently reported slide chamber system (Lindner et al., 2021) was used, which can reproducibly be operated in a simple flow circuit without the need for a complex bioreactor system (Figures 1A–D).

In a preliminary experiment shear stresses in the range of 1.7–10 dyn cm^−2^, mimicking shear in capillaries and veins as well as low arterial shear stress (Ballermann et al., 1998; Paszkowiak and Dardik, 2003), were applied to monolayers cultured on TCPS, inducing the alignment of both HUVECs and iPSC-ECs toward the flow direction (Supplementary Figure S1). In general, this behavior is well known for ECs (Thoumine et al., 1995; Steward et al., 2015) and has been shown previously by several groups for HUVECs cultured under different surface shear stresses (Koo et al., 2013; Li and Wang, 2018; Wang et al., 2020). Additionally, in our hands HUVECs showed a shear and time dependent reaction to flow, as seen by a faster elongation and alignment with higher surface shear. However, the application of 10 dyn cm^−2^ led to a disruption in the cellular monolayer after 96 h. As this level of shear corresponds to arterial conditions, our results imply a limited suitability of vein-originating HUVECs as a model for arterial ECs, at least for dynamic long-term studies. A previous result hinting in this direction was obtained by Gouverneur et al. (2006b) who reported a reduction of HUVEC number already after 24 h of culture with a surface shear stress of 10 dyn cm^−2^.

A review of literature data on the flow-induced alignment of iPSC-ECs reveals contrary results as several groups found a reaction of iPSC-EC orientation to flow (Sivarapatna et al., 2015; Wang et al., 2016; Ingram et al., 2018; Arora et al., 2019; Ciampi et al., 2019; Rosa et al., 2019; Atchison et al., 2020), while others did not see any effect comparable to primary ECs in the absence of cyclosporine A treatment (Tiemeier et al., 2019). Comparing HUVEC behavior under flow to iPSC-ECs in our setup, we not only observed a flow-induced alignment of the latter but also a markedly faster response as regardless of applied shear strength, cells were fully aligned after 24 h (Supplementary Figure S1). This could be explained by iPSC-ECs being in a less mature cellular state without previous contact to fluid flow. The shear naïvety makes the cells potentially more susceptible toward dynamic culture conditions (Sivarapatna et al., 2015; Arora et al., 2019). Additionally, the employed iPSC-ECs were slightly larger in size compared to HUVECs. The larger surface area exposed to the mechanical influence of flow might add to the shortened time needed for cellular alignment.

For the following comparative glycocalyx study we decided on using a physiologically relevant surface shear stress of 6.6 dyn cm^−2^ applied to monolayers, as this condition allows for a fast alignment of not only iPSC-ECs but also HUVECs without negative influence on cellular monolayer integrity. As a study by Koo et al. (2013) demonstrated, the time scale for glycocalyx formation on HUVECs is in the range of 2–3 days. Therefore, we decided on a culture time of 96 h to ensure full maturation of the polysaccharide surface layer. HUVECs and iPSC-ECs cultured on PGE-coated PS surfaces, with prospect of planned cell sheet engineering experiments, showed similar flow-alignment after 96 h as seen before on TCPS, and endothelial differentiation status was preserved for all conditions (Figure 2). Subsequent staining of the glycocalyx components CS, HA and HS revealed some differences between cell types but even more with respect to the culture condition (Figures 3–5). Compared to HUVECs, we found more CS on the surface of the iPSC-EC monolayer under static conditions (Figure 3) as well as a slightly increased amount of HA after static and dynamic culture of iPSC-ECs (Figure 4) while there was no difference in the HS amount between both cell types (Figure 5). Additionally, the glycocalyx thickness was significantly higher in iPSC-ECs compared to HUVECs, although the difference was small as all measured values ranged between ∼1.5 and 2.5 µm (Figure 6). These findings are contrary to findings by Tiemeier et al. (2019), who presented a significantly reduced amount of HA and HS as well as a ∼50% reduced glycocalyx thickness on iPSC-ECs compared to primary ECs after 96 h application of laminar shear stress (5 dyn cm^−2^). An explanation could be the fact that the iPSC-ECs used in their experiments were generated according to a different protocol (Orlova et al., 2014b) than the cells in our study (Olmer et al., 2018). As described by Jang et al. (2019) iPSC-ECs from different origins and differentiation protocols are often hard to compare as their characteristics are heterogeneous. Given that endothelial function relies strongly on glycocalyx functionality, analysis of this important cellular characteristic should find its way into the standard evaluation of iPSC-ECs used as endothelial models. Our findings on glycocalyx properties of the used iPSC-ECs suggest their superior maturity regarding glycocalyx formation.

Furthermore, the comparison of glycocalyx status after static and dynamic culture is important, as it has been shown before that, for example, glycocalyx thickness is highly dependent on applied fluid flow in *in vitro* studies (Ueda et al., 2004; Gouverneur et al., 2006b; Siren et al., 2021). In our present study we also found a significantly higher glycocalyx layer thickness after dynamic culture with ∼2 µm for HUVECs and ∼2.5 µm for iPSC-ECs (Figure 6). These established thicknesses fit with values in the range of 1.7–3 µm obtained for diverse types of ECs in previous *in vitro* studies (Barker et al., 2004; Stevens et al., 2007; Zeng et al., 2013; Tiemeier et al., 2019) and with thicknesses established *in vivo* between 0.5 and 4.5 µm (Reitsma et al., 2007). However, some studies reported unphysiological glycocalyx thicknesses in the range of ∼30 nm (Potter and Damiano, 2008; Chappell et al., 2009). Moreover, thickness determinations after fixation were questioned because of alterations to the glycocalyx *via* dehydration (Curry and Adamson, 2012). Therefore, we additionally performed a native thickness determination using WGA, verifying our aforementioned results (Supplementary Figures S4, S5). When analyzing the glycocalyx composition and potential changes induced by fluid flow we found a slightly increased amount of uniformly distributed CS on HUVECs after dynamic culture (Figure 3), which is somewhat surprising as CS is not a typical mechanosensor (Pahakis et al., 2007; Siren et al., 2021). Nevertheless, also Zeng and Tarbell (2014) found an upregulation of CS after dynamic culture of bovine aortic ECs with a uniform CS distribution. HS and HA on the other hand have previously been described as important mechanosensors of the glycocalyx (Florian et al., 2003; Mochizuki et al., 2003; Ebong et al., 2014). For HA we only saw a reaction to flow in iPSC-ECs, while there was no difference in the amount of HA on the HUVEC surface (Figure 4). However, the increase of HA in HUVECs has been shown by Gouverneur et al. (2006b) as well as Wang et al. (2020). For HS we found the expected strong increase in amount on the surface of the endothelial layer after application of flow (Figure 5), which was also described by Giantsos-Adams et al. (2013) for HUVECs and by several groups for bovine aorta endothelial cells (Florian et al., 2003; Pahakis et al., 2007; Ebong et al., 2014). HS generally shows the strongest reaction of the three important GAGs contained in the glycocalyx toward flow as it is, amongst other binding partners, attached to syndecans. These are transmembrane proteins comprising several functions, for example, the transduction of mechanical influences on the cell surface into the interior by interaction with the cytoskeleton *via* their cytoplasmic tail region (Giantsos-Adams et al., 2013; Tarbell et al., 2014). The dense outstretched mesh structure with formation of thicker HS fibers shown in Figure 5 is in line with the appearance of HS layers on rat fat pad endothelial cells (Zeng and Tarbell, 2014) and human umbilical vein smooth muscle cells (Kang et al., 2017).

To assess the functional integrity of the glycocalyx we employed a leucocyte adhesion assay as for a mature glycocalyx in the physiological state the polysaccharide moiety sufficiently covers cellular adhesion molecules, thereby preventing the adhesion and subsequent extravasation of blood cells (Kalucka et al., 2017). Despite the increase of adhesion molecule expression (ICAM-1) after application of laminar flow, Siren et al. (2021) demonstrated a reduced leucocyte adhesion correlated to an increased glycocalyx thickness. After glycocalyx shedding or in the case of a thin immature glycocalyx, leukocytes are able to interact with the EC layer and subsequently adhere (Kalucka et al., 2017). Performing a simple static leucocyte adhesion assay, we could show a significant reduction in PBMC binding to the endothelial surface after dynamic culture for both cell types, arguing for a more mature glycocalyx layer compared to their status after static culture. This is in line with the thickness improvement identified after application of fluid flow and demonstrates functionality. Similar results were obtained by Delgadillo et al. (2020) for HUVECs and neutrophils, showing higher immune cell adhesion for lower surface shear stresses.

Subsequent to the extensive analysis of cellular directionality and glycocalyx properties we aimed to utilize the aligned cellular monolayers comprising a functional glycocalyx for cell sheet engineering with the prospect of fabricating 3D vessel structures. As one of the mayor obstacles of 3D tissue engineering is the inefficient vascularization of fabricated constructs, vascular tissue engineering has been in the focus for decades (Devillard and Marquette, 2021). Several advances have been made toward perfusable 3D vessel formation, ranging from the inner lining of tubular constructs using ECs (Weinberg and Bell, 1986; Villalona et al., 2010) to promising advances employing cell sheet engineering to form 3D vascular grafts (L'Heureux et al., 1998; Kubo et al., 2007; Rayatpisheh et al., 2014; Othman et al., 2015). In a first proof-of-concept, we aimed to translate the advantages of EC culture under physiological flow toward tissue engineering applications of blood vessel mimics. Therefore, we cultured HUVECs and iPSC-ECs under static and dynamic conditions on thermoresponsive cell culture substrates (Stöbener et al., 2018; Stöbener and Weinhart, 2020) and at first successfully detached intact monolayers after a static culture time of 96 h simply by temperature reduction. To our knowledge, this is the first generation of a human iPSC-EC cell sheet, as this technique has only yet been employed using mouse iPSC-ECs (Hibino et al., 2012). For dynamically cultured cells, we observed aligned HUVEC sheet detachment but no complete self-detachment of iPSC-EC sheets (Figure 8). Looking for an explanation of sheet disruption, we verified the integrity of cell-cell contacts within the confluent monolayers for all conditions and found ZO-1 and VE-cadherin homogenously distributed to cell-cell contact sites (Figure 9A), as shown before, for example, for iPSC-ECs and VE-cadherin (Olmer et al., 2018) or HUVECs and ZO-1 (Bartosova et al., 2021). The observed nuclear staining of ZO-1 for all conditions can be explained by the still dynamic remodeling of cell-cell contacts after 96 h of culture, leading to a nuclear localization of ZO-1 (Gottardi et al., 1996). Furthermore, staining of actin and vinculin revealed a pronounced generation of stress fibers and focal adhesions after application of fluid flow in HUVECs and iPSC-ECs (Figure 9B, Supplementary Figures S9), which is a typical EC reaction to surface shear stress (Franke et al., 1984; Thi et al., 2004; Katoh et al., 2008). As both cell types showed a similar reorganization of the cytoskeleton upon dynamic culture, we conclude, consistent with data shown on cellular alignment and glycocalyx formation, that the iPSC-ECs are comparably mature as the primary HUVECs in terms of mechanosensing. However, the findings on cell-cell interactions as well as state of cytoskeleton and focal adhesion cannot explain the deficit in self-detachment of iPSC-ECs as confluent layer. One difference to HUVEC culture is the pre-coating using fibronectin in the case of iPSC-ECs. Potentially, increased presence of this extracellular matrix glycoprotein can mediate stronger interaction of focal adhesions with the underlying substrate, thereby impeding self-detachment of the monolayer.

To increase the chance of intact detachment of an aligned iPSC-EC sheet, we delivered mechanical support by simultaneous transfer of the sheet during detachment to a tubular 3D-printed scaffold (Figure 10). By this preliminary experiment we could show that rolling of a flow-aligned iPSC-EC sheet onto a tube with preservation of cellular orientation is possible. Some groups have previously reported the wrapping of aligned HUVEC sheets (Rayatpisheh et al., 2014; Othman et al., 2015) after thermally triggered sheet detachment from micropatterned surfaces, which induced the cellular alignment. However, there have not been any studies so far on 3D vessel engineering of flow-aligned HUVEC or iPSC-EC sheets comprising functional mature glycocalyx. After these promising initial results, future studies will focus on the implementation of an aligned tubular sheet construct into a dynamic flow circuit as described by Elomaa et al. (2022).

In conclusion, we were able to demonstrate the capability of HUVECs and iPSC-ECs to respond to physiological fluid flow and show its beneficial effect on glycocalyx formation and integrity compared to static culture. With the help of thermoresponsive substrates, we were able to generate either self-detached cell sheets or a 3D construct comprising a rolled sheet with preservation of the flow-induced cellular alignment. We envision our results to be valuable contributions for the improvement of endothelial *in vitro* models in the future.

## Data Availability

The raw data supporting the conclusion of this article will be made available by the authors, upon reasonable request.
