# Supplementary material for: Flow-induced glycocalyx formation and cell alignment of HUVECs compared to iPSC-derived ECs for tissue engineering applications
Source: Front Cell Dev Biol. 2022 Sep 5;10:953062. doi: 10.3389/fcell.2022.953062 (PMC9483120; doi:10.3389/fcell.2022.953062)
Supplement: Supplementary file 1 [file DataSheet1.pdf]

Supplementary Material – Lindner et al. 2022

Outline

1 Correlation of shear stress and orientation of the cells 2

2 Directionality analysis 4

3 Evaluation of native glycocalyx thickness 6

4 Evaluation of the PBMC adhesion assay 8

5 Cell sheet detachment after 48 h 10

6 Formation of stress fibers due to fluid flow 11

7 References 12

1 Correlation of shear stress and orientation of the cells

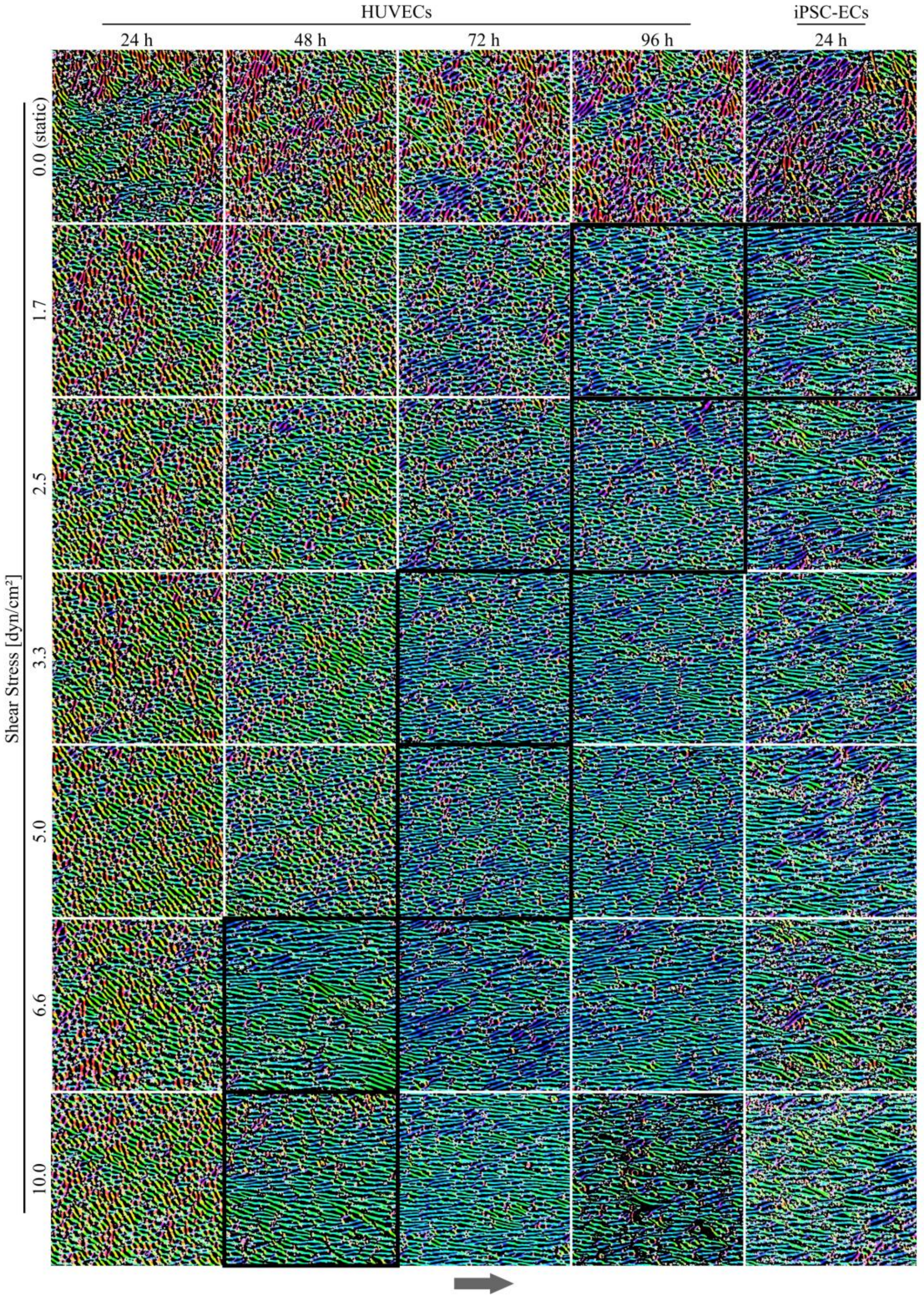

Supplementary Figure S1. Time and shear stress dependent orientation of HUVECs and iPSC-ECs displayed as color maps. Black frames highlight the first timepoint comprising an apparent orientation and elongation of the respective cells with the fluid flow. (Arrow indicates flow direction. Image size: 670x670 µm, n=1).

To define optimal parameters for alignment and elongation of endothelial cells (ECs) under flow conditions, HUVECs were seeded on ibidi µ-slides (channel heights 0.2 and 0.4 mm) and cultured under different volume flow rates. Various shear stresses in a physiological range from 0 (static culture) to 10 dyn cm^{-2} were applied in a preliminary screening for up to 96 h. After 48 h, cellular alignment and elongation was already observed for ≥6.6 dyn cm^{-2}, whereas cells cultured with 3.3 to 5 dyn cm^{-2} required 72 h for alignment. Alignment and elongation were observed at ≥1.7 dyn cm^{-2} after 96 h. High shear stress conditions (10 dyn cm^{-2}) resulted in a reduction of cellular confluency after 96 h, as indicated by black areas. Based on these results, we finally decided to apply 6.6 dyn cm^{-2} throughout the remaining experiments, which at the same time yields a fast alignment without being apparently harmful to cells for ≥96 h. Adaption of the experiment to iPSC-ECs demonstrated their high capacity for alignment and elongation, as the cells were (already) aligned after 24 h under all flow conditions.

2 Directionality analysis

To qualitatively analyze and visually highlight the influence of varying culture conditions on the orientation of ECs as already shown in section 1 of this supplementary material, phase contrast images of ECs after static and dynamic culture were pre-processed using *ImageJ* and subsequently an orientation/angle-specific color coding was added using the *OrientationJ* plugin [1]. For pre-processing, the background was subtracted from the raw phase contrast images (rolling ball, radius 100 px), a Gaussian filter (σ = 1 µm, scaled), and a bandpass filter (down to 20 µm, no lower limit) were applied to compensate for inhomogeneous illumination and smoothen the image [2]. The image is binarized via (auto)-thresholding according to Huang *et al.* [3], and finally processed/colored using the *OrientationJ* plugin (local window: σ = 3 pixel). Color map is showing hue: orientation, saturation: coherency and lightness of the original image.

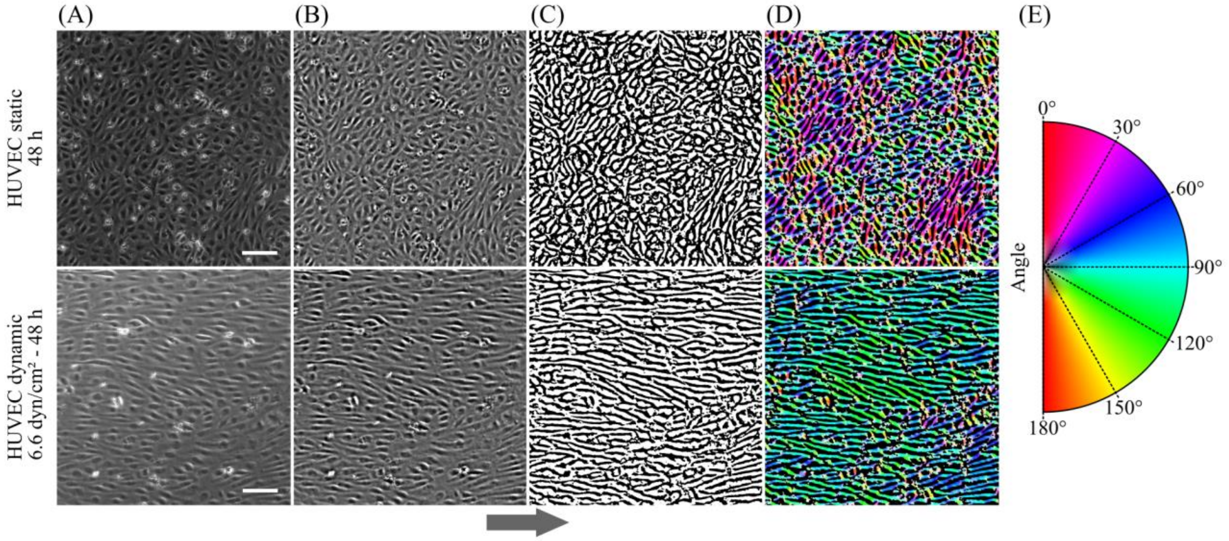

Supplementary Figure S2. Image processing steps for qualitative directionality analysis. **(A)** Original phase contrast images from HUVECs cultured under static and dynamic conditions for 48 h. **(B)** Preprocessing of the images by background subtraction as well as addition of gaussian blur and bandpass filters to remove uneven illumination and smoothen the image. **(C)** Thresholding to create a binary image. **(D)** Generation of color-map using the *OrientationJ*-plugin in *ImageJ*, adding an angle-dependent color to cell borders illustrated in **(E)**. (Arrow indicates flow direction. Scale bars: 100 µm).

ImageJ was further used to quantify the effect of culture conditions on cellular alignment/orientation. Initially, the background of phase contrast images was subtracted (rolling ball, radius = 100 pixels) and the contrast of images was increased via gamma correction (γ = 0.8) and contrast stretching (*ImageJ*'s *enhance contrast* function, cutoff parameter = 0.7% saturated pixels). Using the *find maxima* function (prominence = 25, excluded on edges, light background), the cell centers were determined and the image was segmented into single cell areas accordingly. The *Extended Particle*

Analyzer of the *BioVoxxel* toolbox was used to determine/measure the main orientation of single cells[1] [4].

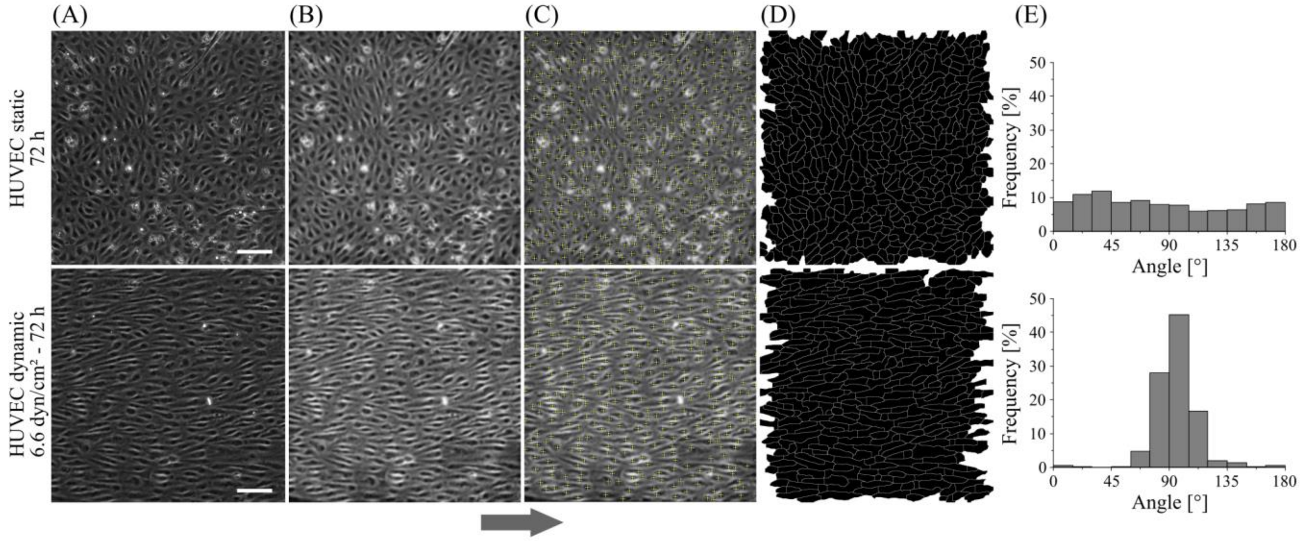

Supplementary Figure S3. Image processing steps for quantitative directionality analysis. **(A)** Original phase contrast images from HUVECs cultured under static and dynamic conditions for 72 h. **(B)** Preprocessing of the images by background subtraction and gamma/brightness adjustment to enhance contrast. **(C)** Application of the 'Find maxima' function in *ImageJ* and **(D)** segmentation of the images to create a mask of single cells. **(E)** Histogram of main orientation of single cells obtained using the extended particle analyzer in *ImageJ*. (Arrow indicates flow direction. Scale bars: 100 µm).

[1] These parameters were determined empirically and were valid/applicable for all acquired images.

3 Evaluation of native glycocalyx thickness

To estimate glycocalyx thickness(es) under native, non-fixed conditions, ECs after static culture were stained with wheat germ agglutinin (WGA, labelled with Alexa™ 555, ThermoFisher) as well as CMFDA and subsequently imaged via confocal microscopy. Due to the behavior of thermoresponsive polymers and despite using an incubation chamber as well as rapid sample preparation, unfixed cells on PGE-coated substrate started to detach during image acquisition (data not shown). The experiment was therefore performed on tissue culture-treated polystyrene slides (TCPS). To exclude any influence of the cell culture substrate on glycocalyx expression, HUVECs and iPSC-ECs were seeded on TCPS and PGE-coated PS, stained with WGA and imaged via confocal microscopy (after fixation with PFA). As demonstrated in **Supplementary Figure S4**, no difference in glycocalyx expression as indicated by mean fluorescence intensity of WGA-staining was observed between both substrates for each cell type, respectively.

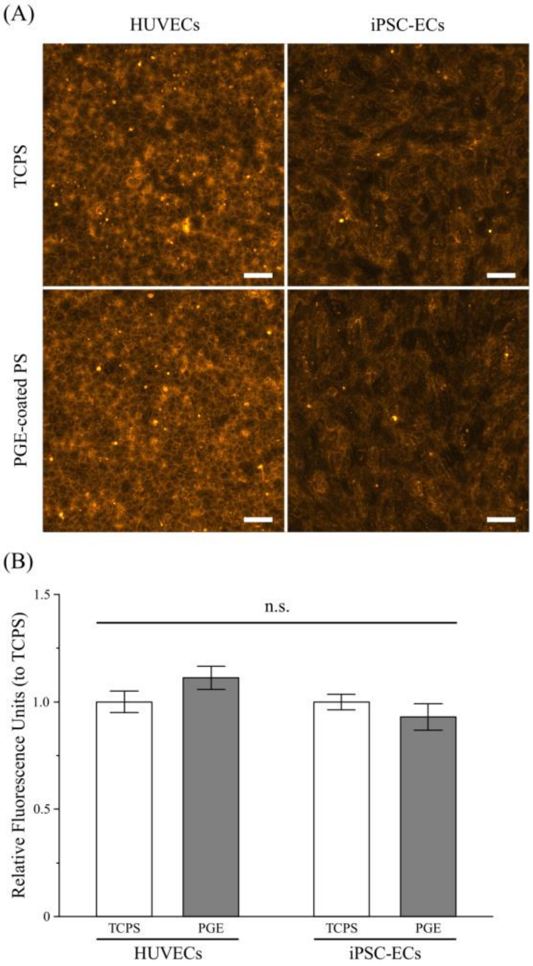

Supplementary Figure S4. Influence of culture substrate on WGA-staining of HUVECs and iPSC-ECs under static conditions (96 h) **(A)** Representative confocal image of lectin-staining (orange) on cells cultured on tissue culture and PGE-coated polystyrene. **(B)** Quantification of fluorescence intensities. Data shown as mean fluorescence units relative to the corresponding TCPS sample. No significant differences were found between the TCPS and PGE-coated PS culture substrates (Mann-Whitney Test, n=3) (Scale bars: 100 µm).

Subsequently, the glycocalyx thickness of non-fixed endothelial cells was estimated via confocal microscopy using Airyscan super-resolution mode. Orthogonal projections of Z-stacks demonstrate the native glycocalyx thickness being in the range of a few micrometers (2-4 µm), as shown in **Supplementary Figure S5**.

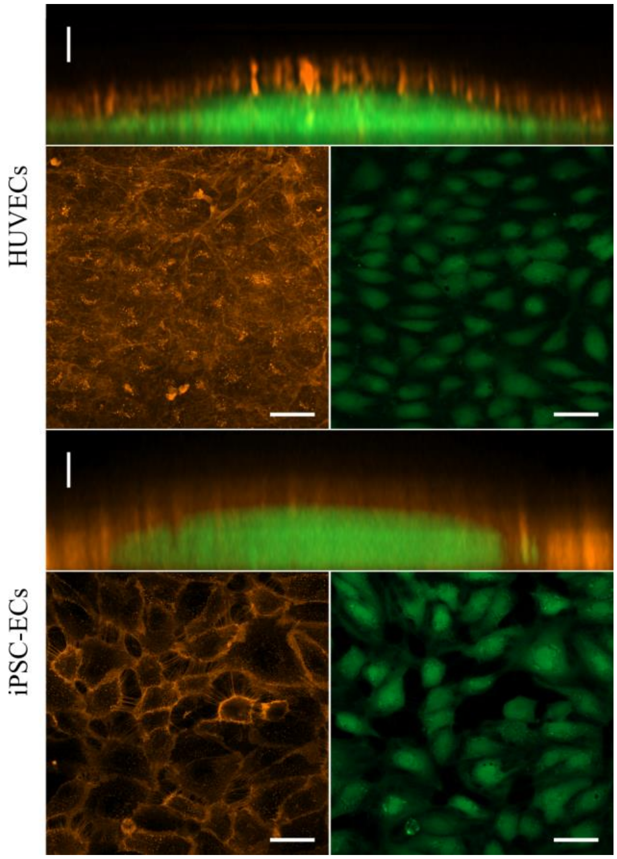

Supplementary Figure S5. Estimation of glycocalyx thickness under native conditions. Orthogonal projection and confocal images of HUVECs and iPSC-ECs after static culture for 96 h stained with WGA (glycocalyx, orange) and CMFDA (cytosol, green) under unfixed conditions. Scale bars: horizontal 50 µm, vertical 2.5 µm).

4 Evaluation of the PBMC adhesion assay

A peripheral blood mononuclear cell (PBMC) adhesion assay was established to investigate glycocalyx functionality. **Supplementary Figure S6A** illustrates the processing of the raw data and analysis. HUVECs were incubated with fluorescently labeled PBMCs, washed, fixed and subsequently imaged using fluorescence confocal microscopy. The images were processed with *ImageJ* (conversion from 16 to 8 bit, application of a gaussian filter, σ = 1 µm and manual thresholding ranging from 30 to 255) and analyzed using *ImageJ's Particle Analyzer*, counting the number of PBMCs per image (size: 30-125 pixel^2).

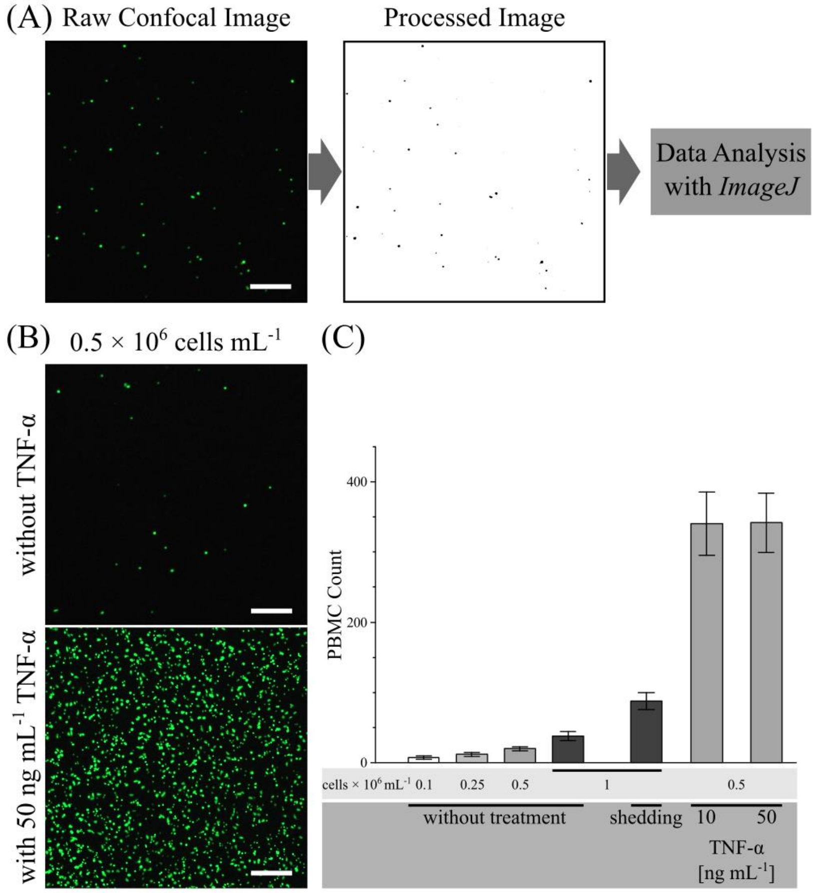

Supplementary Figure S6. Establishment of PBMC-assay to assess glycocalyx functionality. **(A)** Processing of the raw confocal image showing CMFDA-labelled PBMCs in green, by thresholding to create a binary image. The processed image was analyzed via *ImageJ particle analyzer* to obtain PBMC counts. (Scale bar: 200 µm) **(B)** Representative confocal images demonstrating the impact of a TNF-α-incubation (positive control) on PBMC adhesion to HUVECs after 96 h of static culture (Scale bars: 200 µm) **(C)** Influence of cell number as well as shedding of the glycocalyx (mechanical, TNF-α incubation) on adhesion of PBMCs to HUVEC monolayers after 96 h of static culture. Data are shown as mean ± SEM (n=1).

Various PBMC concentrations were tested and ECs were differentially treated to validate the assay. As a positive control, HUVECs were incubated for 6 hours with 10 or 50 ng mL^{-1} of the

proinflammatory cytokine TNF-α, which is known to increase leukocyte adhesion to ECs via upregulation of adhesion molecules (CAMs), and concomitant shedding of their glycocalyx [5, 6]. In addition, a concentration-dependent increase in adherent PBMCs was demonstrated. Finally, an apparent increase in PBMC adhesion after mechanical disruption of the glycocalyx via excessive pipetting demonstrated the assay's suitability to examine glycocalyx functionality.

Subsequently, an analysis of PBMC adhesion to HUVECs and iPSC-ECs after static culture on PGE-coated PS as culture substrate was conducted. Results demonstrated no significant difference in leucocyte count on the monolayer surface for both cell types (**Supplementary Figure S7**).

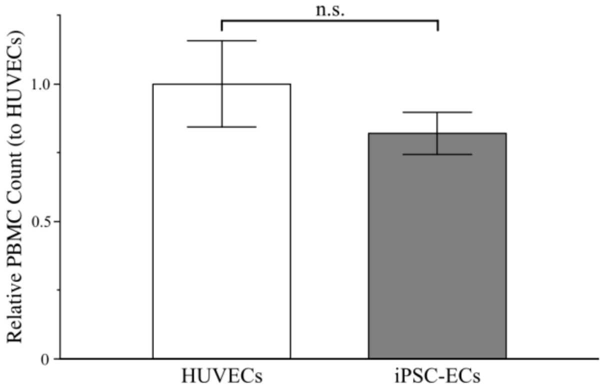

Supplementary Figure S7. Comparison of relative PBMC adhesion to HUVECs and iPSC-ECs after 96 h of static culture. Relative data are presented as mean ± SEM with respect to "HUVECs static" (n=4, Mann-Whitney Test).

5 Cell sheet detachment after 48 h

As previously shown in **Supplementary Figure S1**, the application of 6.6 dyn cm^{-2} for 48 h is sufficient for a pronounced alignment of both, HUVECs and iPSC-ECs on TCPS. Since one objective of this study was to generate confluent cell sheets of different endothelial cells, both under static (non-aligned) and dynamic (aligned) culture conditions, we attempted to detach cell sheets from PGE-coated PS first after 48 h. **Supplementary Figure S8A** demonstrates an elongation and alignment of HUVECs and iPSC-ECs on PGE-coated PS comparable to TCPS. By transferring the slides into room temperature PBS^{-}, we triggered singularization and partial detachment of ECs under all culture conditions (**Supplementary Figure S8B**).

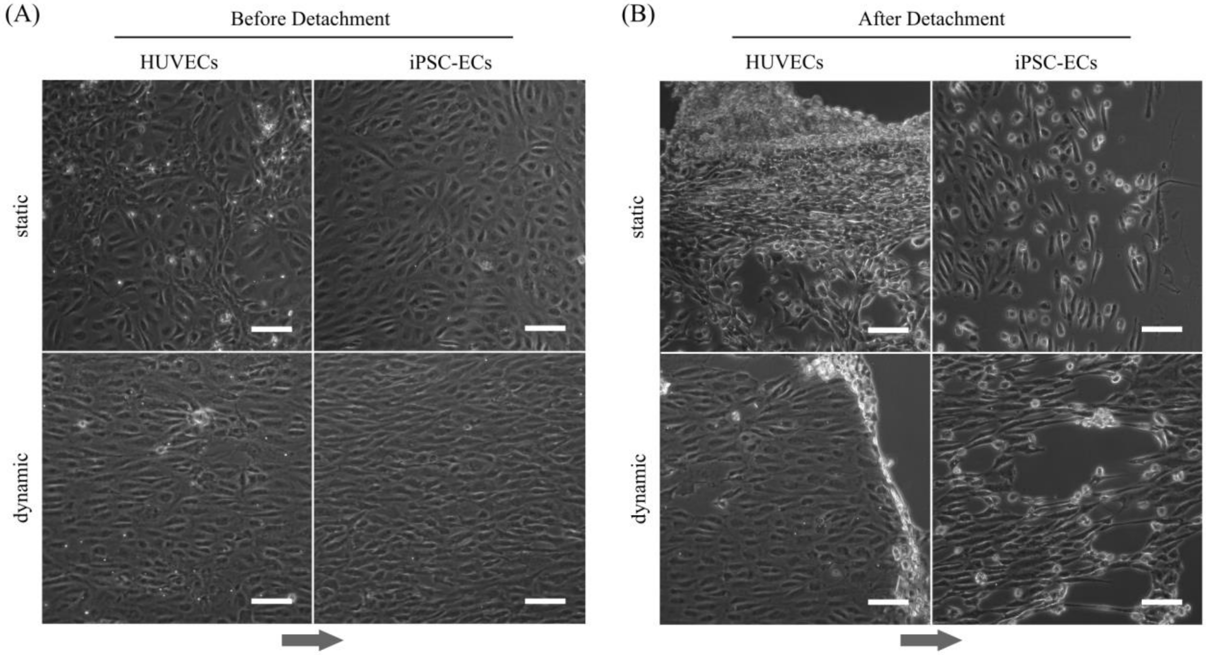

Supplementary Figure S8. Detachment of HUVECs and iPSC-ECs after 48h of static or dynamic culture. Phase contrast images were taken **(A)** before and **(B)** after (incomplete) detachment. (Arrow indicates flow direction. Scale bars: 100 µm, n=2).

6 Formation of stress fibers due to fluid flow

The application of shear stress to ECs results in the formation of stress fibers, which are known to increased cellular adhesion to the underlying substrate [7]. **Supplementary Figure S9** displays confocal images of endothelial focal adhesions after static and dynamic culture conditions, stained with phalloidin (red) and vinculin (green), demonstrating the excessive formation of stress fibers after exposure to shear stress.

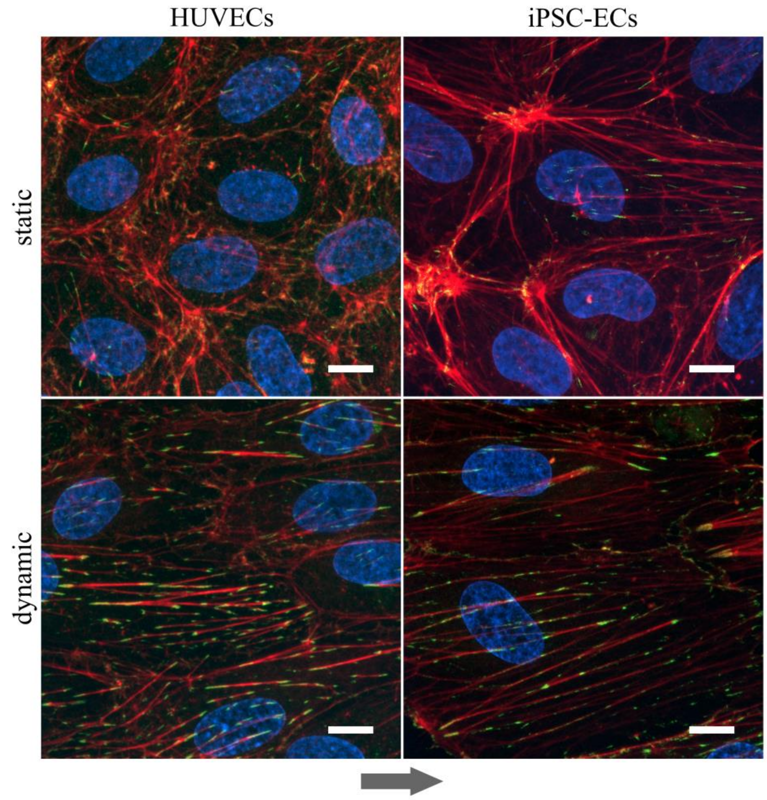

Supplementary Figure S9. Confocal images showing focal adhesions after static and dynamic culture of HUVECs and iPSC-ECs for 96 h. Cells were stained for phalloidin (red) and vinculin (green). Nuclei were counterstained with Hoechst 33342 (blue). (Arrow indicates flow direction. Scale bars: 10 µm).

7 References

1. Rezakhaniha, R.; Agianniotis, A.; Schrauwen, J.T.; Griffa, A.; Sage, D.; Bouten, C.V.; van de Vosse, F.N.; Unser, M.; Stergiopulos, N. Experimental investigation of collagen waviness and orientation in the arterial adventitia using confocal laser scanning microscopy. *Biomech. Model. Mechanobiol.* **2012**, *11*, 461-473; DOI:10.1007/s10237-011-0325-z.
2. Xu, F.; Beyazoglu, T.; Hefner, E.; Gurkan, U.A.; Demirci, U. Automated and adaptable quantification of cellular alignment from microscopic images for tissue engineering applications. *Tissue Eng. Part C Methods* **2011**, *17*, 641-649; DOI:10.1089/ten.tec.2011.0038.
3. Huang, L.-K.; Wang, M.-J.J. Image thresholding by minimizing the measures of fuzziness. *Pattern Recognition* **1995**, *28*, 41-51; DOI:10.1016/0031-3203(94)e0043-k.
4. Brocher, J. "The BioVoxxel image processing and analysis toolbox." Paper presented at the European BioImage Analysis Symposium 2015.
5. Mattila, P.; Majuri, M.L.; Mattila, P.S.; Renkonen, R. TNF alpha-induced expression of endothelial adhesion molecules, ICAM-1 and VCAM-1, is linked to protein kinase C activation. *Scand. J. Immunol.* **1992**, *36*, 159-165; DOI:10.1111/j.1365-3083.1992.tb03087.x.
6. Chappell, D.; Hofmann-Kiefer, K.; Jacob, M.; Rehm, M.; Briegel, J.; Welsch, U.; Conzen, P.; Becker, B.F. TNF-alpha induced shedding of the endothelial glycocalyx is prevented by hydrocortisone and antithrombin. *Basic Res. Cardiol.* **2009**, *104*, 78-89; DOI:10.1007/s00395-008-0749-5.
7. Franke, R.-P.; Gräfe, M.; Schnittler, H.; Seiffge, D.; Mittermayer, C.; Drenckhahn, D. Induction of human vascular endothelial stress fibres by fluid shear stress. *Nature* **1984**, *307*, 648-649.
